# Role of Defects, Pores, and Interfaces in Deciphering the Alkali Metal Storage Mechanism in Hard Carbon

**DOI:** 10.1021/acsaem.2c02591

**Published:** 2022-12-29

**Authors:** Alexandros Vasileiadis, Yuqi Li, Yaxiang Lu, Yong-Sheng Hu, Marnix Wagemaker

**Affiliations:** † Storage of Electrochemical Energy, Department of Radiation Science and Technology, Faculty of Applied Sciences, Delft University of Technology, Mekelweg 15, Delft2929JB, The Netherlands; ‡ Key Laboratory for Renewable Energy, Beijing Key Laboratory for New Energy Materials and Devices, Beijing National Laboratory for Condensed Matter Physics, Institute of Physics, Chinese Academy of Sciences, Beijing100190, China; § College of Materials Science and Optoelectronic Technology, University of Chinese Academy of Sciences, Beijing100049, China

**Keywords:** hard carbon, sodium-ion battery, alkali metal storage mechanism, nanopores, lithium-ion battery

## Abstract

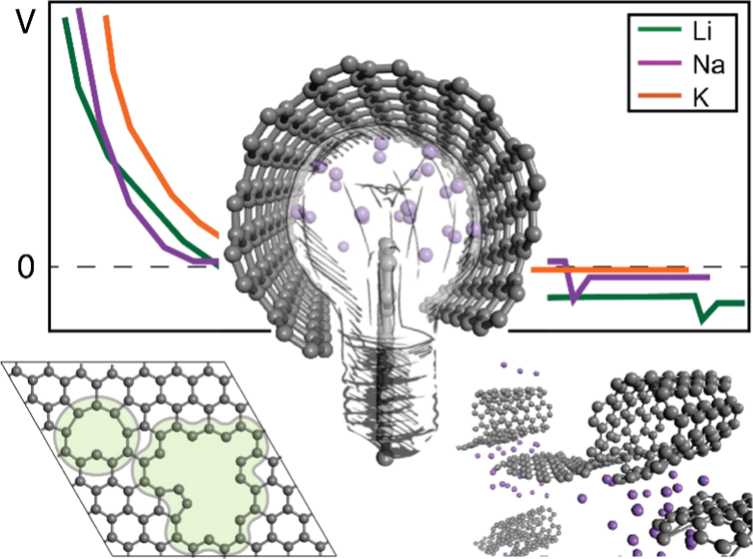

There are several questions and controversies regarding the Na storage mechanism in hard carbon. This springs from the difficulty of probing the vast diversity of possible configurational environments for Na storage, including surface and defect sites, edges, pores, and intercalation morphologies. In the effort to explain the observed voltage profile, typically existing of a voltage slope section and a low-voltage plateau, several experimental and computational studies have provided a variety of contradicting results. This work employs density functional theory to thoroughly examine Na storage in hard carbon in combination with electrochemical experiments. Our calculation scheme disentangles the possible interactions by evaluating the enthalpies of formation, shedding light on the storage mechanisms. Parallel evaluation of the Li and K storage, and comparison with experiments, put forward a unified reaction mechanism for the three alkali metals. The results underline the importance of exposed metal surfaces and metal–carbon interfaces for the stability of the pore-filling mechanism responsible for the low-voltage plateau, in excellent agreement with the experimental voltage profiles. This generalized understanding provides insights into hard carbons as negative electrodes and their optimized properties.

## Introduction

The energy transition toward renewables and electric transport has introduced new standards and requirements for next-generation storage applications, challenging the research community to realize batteries with better capabilities, safety, and environmental benignity. Li-ion batteries (LIBs) play a central role in portable electronic devices and electric vehicles, offering superior energy and power densities among the available battery technologies.

However, it is questionable whether LIBs can accommodate the expanding requirements of large-scale applications due to the high cost and the limited distribution of lithium resources.^1,2^ Na-ion batteries (NIBs) are widely considered promising alternatives, especially for renewable energy and grid stabilization applications, taking advantage of sodium’s abundance, accessibility, and inexpensive electrolyte salts.^3−5^ Further advancement of NIBs heavily relies on developing and optimizing innovative negative electrodes, posing the main bottleneck in achieving superior performances.^6,7^ This necessity originates from the inability of graphite, the most commercially successful negative electrode for LIBs, to store Na ions between the highly ordered graphene layers of its microstructure.^8−10^

One well-investigated anode option that has drawn vast scientific interest as an efficient alternative within the carbon family is hard carbon (HC).^8,11,12^ Unlike graphite, HC can store Na ions, offering stable cycling and large specific capacities that can exceed 300 mAh/g.^11,13^ Further, HC can be derived from precursors originating in a wide variety of low-cost biomass products such as wood, peat moss, lignin, and banana leaves, thus potentially meeting the sustainability prerequisites of environmental friendliness and economic viability.^8,14,15^ Precursors with high sp^2^ hybridization, such as aromatic petroleum derivatives, will form soft carbon with a relatively regular carbon content after carbonization, while precursors with high sp^3^ carbon content, such as cellulose and plastics, generally form disordered structures of hard carbon.^15^

Structurally, HC is rather complicated, differing from graphite, a near-perfectly ordered arrangement of graphene sheets 3.3 Å apart. High mechanical hardness limits the flattening of the graphene sheets and the capability to graphitize.^8,16^ As a result, HC is often described as a mixture of curved and entangled graphene and fullerene-like structures.^8,16−18^ This turbostatic network creates nanopores in a variety of sizes and number distributions.^13^ The graphene sheets are highly defective, containing single and double vacancies, heteroatom contamination, and a small concentration of pentagon and heptagon carbon rings that enable sheet bending.^16,17,19,20^ Some stacking of the graphene sheets is observed, usually ranging from two to five layers with expanded interlayer distances (3.7–4.0 Å) compared to graphite.^18,21,22^ The ratio of geometrical characteristics mentioned above can be tuned with the synthesis process, for example, the annealing temperature, and thus adjust electrochemical performance.^13,21^

**Figure 1:**
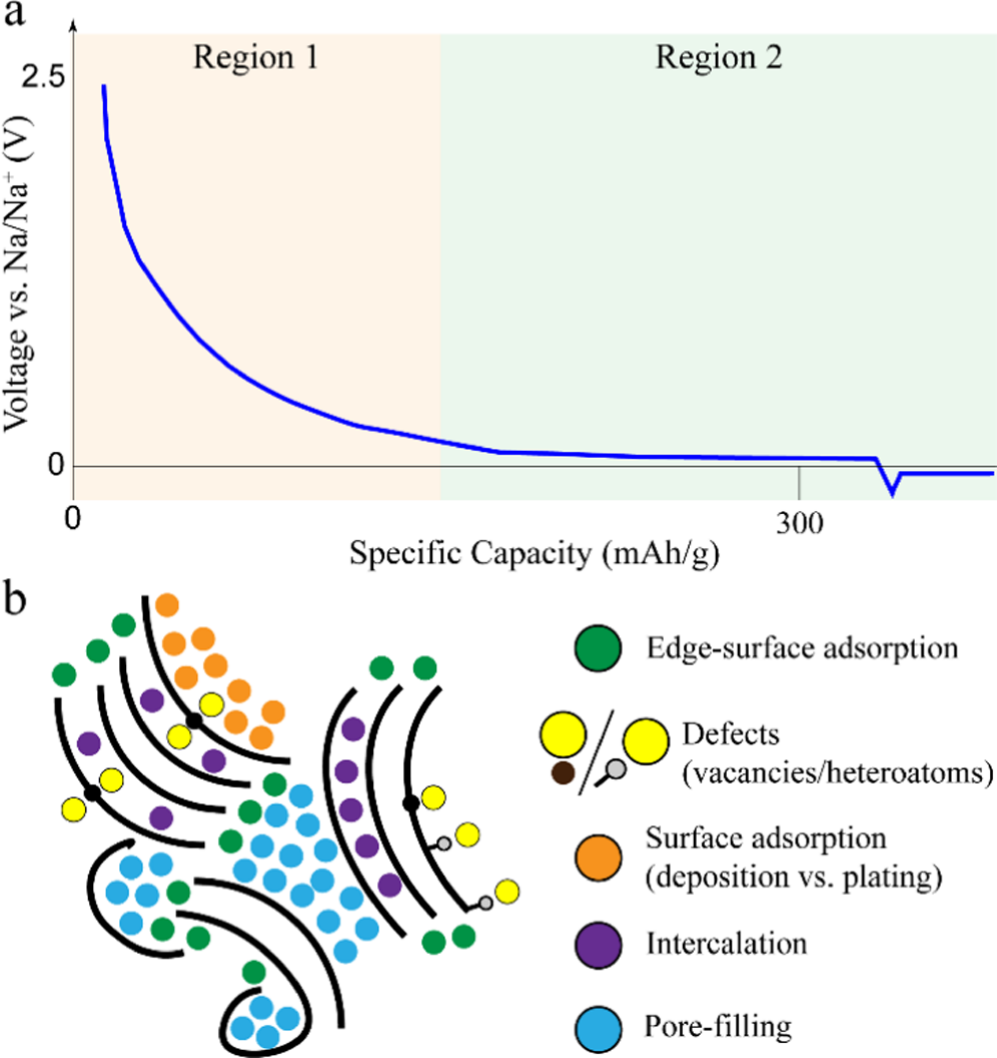
(a) Typical Na–HC voltage profile referenced to Na metal. We can distinguish the high-voltage sloping (region 1) and the low-voltage plateau (region 2) regions. The voltage profile concludes in a V-shaped curve, initiating the Na metal plating when the maximum capacity of HC is achieved. (b) Schematic representation of the HC structure (black curved lines) and Na on the variety of binding positions according to the available reaction mechanisms (colored circles).

The vast amount of discrete morphological features creates a plethora of redox options for the incoming alkali-metal ion. A typical Na–HC electrochemical voltage curve is presented in Figure 1, along with a schematic illustration of the HC structure and the available reaction mechanisms. The voltage profile generally includes two distinct features, a high-voltage sloping region and a low-voltage plateau. The shape of the voltage profile, including the length of each region that determines the available capacity, is highly dependent on the structural characteristics. This structural dependence provides indirect insights into the electrochemical performance. For example, defect concentration will decrease as the carbonization temperature increases, diminishing capacity from the slope region. In contrast, the nanopore number and size will increase with increasing annealing temperature, enhancing capacity from the low-voltage plateau. Generally, annealing temperatures up to 1100 K lead to HC with significant defects and a dominating slope region.^13^ The sodiation process reaches a “V”-shaped voltage feature, attributed to the sodium nucleation barrier that initiates the plating process.

Despite intensive research, fundamental questions remain, especially concerning the sodium storage mechanism and how it relates to the HC’s structural characteristics.^8,11,13^ This link is critical for electrochemical performance optimization, enabling the full potential of NIBs.

The first descriptive model developed to characterize the electrochemical storage mechanism of Na ions in HC was established by Stevens and Dahn,^18^ who performed *in situ* small-angle X-ray scattering (SAXS) experiments. Broadening of the peaks reflecting the interlayer graphene distance was initially detected, followed by a reduction in the electron density contrast between the pores and the carbon matrix. Consequently, an “intercalation–pore filling”^11,18^ sodiation mechanism was put forward describing the sloping and the plateau regions of the sodiation voltage profile, respectively. Komaba et al.^23^ confirmed the above picture, observing similar fluctuations in the electron density contrast at the latter sodiation stages that respond to Na clustering in nanopores. Moreover, an operando Raman spectroscopy study^24^ argued in favor of the intercalation–pore filling mechanism by detecting reversible changes in the G and D graphitic Raman peaks linked to intercalation and a constant-energy G peak over the plateau region linked to Na filling the nanopores.

Contrarily, Bai et al.^25^ utilized Raman spectroscopy to probe possible shifts in the G-band, a characteristic feature of Na intercalation in HC. However, no such shift was detected, ruling out the intercalation mechanism. The above observation has been additionally supported by X-ray diffraction (XRD) experiments,^26^ where the d-spacing remained constant during cycling. Instead, a correlation between the defect concentration of HC and the sloping region’s reversible capacity was observed, pointing toward an adsorption mechanism.^25^ Further, filling the pores with sulfur, which is expected to hinder the pore-filling mechanism, effectively diminished the low-voltage capacity. As a result, an “adsorption–pore filling” model was put forward,^25^ where Na initially resides in the vicinity of defect sites, edge surfaces, and heteroatoms (mostly N and O), followed by Na insertion into the micro/nanopores. Several other groups^26−29^ supported this model by correlating their electrochemical capacity results with the level of order in their HC samples. In some cases, the high-voltage region (region 1) is broken down into two distinct stages, where defect adsorption occurs at higher potentials (stage 1), followed by Na adsorption in isolated graphene sheets (stage 2).^26^

Nuclear magnetic resonance (NMR) results^28,30^ are on the same page, probing peak shifting in the plateau region, a charge transfer signal toward metallic Na, without fully reaching it. This observation is evidence of semimetallic Na filling the hard-carbon pores at the latter sodiation stages. Furthermore, solid-state NMR and pair distribution function (PDF) analysis revealed that the formation of metallic Na clusters with diameters in the range of 1.3–1.5 nm is responsible for the capacity in the voltage plateau.^30^

Density functional theory (DFT) studies^29^ have also supported the Na pore-filling mechanism by establishing that Na aggregation in small (triangle) clusters is the preferable configuration upon Na dissipation on a graphene sheet, indirectly indicating Na clustering inside a pore. Further, DFT has directly predicted that it is energetically favorable to form three to four Na layers within the graphitic layers, supporting that semimetallic Na cluster formation within the graphitic layers is the origin of the voltage plateau.^31^ Furthermore, DFT computations combined with machine learning on nongraphitic carbon models analyzed the local environment of the inserted Na.^32^ Maximum charge transfer was observed at the initial stages of sodiation, where ionic Na resides near vacancies on the sp^2^ plane. On the other hand, only partially ionic Na was detected for Na pore clustering, with charge transfer between 0.2 and 0.6, producing a voltage plateau.

On the other hand, several research studies argue that Na intercalation is dominant in the low-voltage plateau region.^21,33,34^ Pair distribution function (PDF) analysis^28^ has depicted significant turbostatic disorder in the carbon sheets, while transition electron microscopy (TEM) experiments^35^ have directly observed curved graphene layers and misaligned graphene sheets with wide interlayer distances (0.37–0.41 nm), which are considered sufficient to accommodate Na ions.^34^ Hard-carbon samples with large average graphene interlayer spacing (0.374, 0.395 nm) have been prepared and tested with XRD, and Raman spectroscopy.^21,33,36,37^ During cycling and within the low-voltage region, an increase of the interlayer spacing was observed (0.386, 0.415 nm) suggesting Na intercalation. This is additionally supported by detecting volume expansion at the low-voltage region.^35^ Similar conclusions are drawn by correlating the electrochemical results with the structural characteristics of the HC samples.^17,38^ More specifically, Lu et al.,^38^ who used ball milling to tune the hard-carbon microstructure toward higher exposed surfaces and larger micropores, observed that larger micro-pore volume does not necessarily translate into higher plateau capacities. Moreover, Li et al.^17^ utilized various dopants, introducing a double effect on the HC structures. First, adsorption sites are created, enhancing the slope voltage region, and second, the dopants widen the interlayer distances between the graphene sheets enabling Na intercalation. The above results point toward an “adsorption–intercalation” model, where Na initially resides on the active surface sites near defects, which offer a wide distribution of adsorption energies, followed by Na intercalating between the graphene layers. Generally, curved graphene sheets with distances in the 0.37–0.38 nm range were assumed to be appropriate to facilitate intercalation in a manner that closely resembles the behavior of the two-phase intercalation reaction observed in the lithium counterpart.^21^

Further evidence on the adsorption–intercalation model was provided by DFT studies,^36,39,40^ pointing toward intercalation for the low-voltage plateau. Calculations of Na intercalation in defective and hydrogen-terminated graphene bilayers predict a positive voltage for Na intercalation in a relatively flat voltage plateau just above 0 V (reaching the Na_1_C_8_ phase). The calculated interlayer distance was determined at 0.45 nm for the defective and 0.47 nm for the hydrogen-terminated bilayer. However, the DFT predicted values are considerably higher than those previously considered (0.37–0.38 nm) as sufficient for Na intercalation.

Several studies suggest more complex mechanisms, where the voltage profile is divided into more than two regions. Bommier et al.,^37^ utilizing galvanostatic intermittent titration technique (GITT) experiments, revealed a sharp change in the latter stages of sodiation (0–0.05 V range). Based on this, it was assumed that the sodiation mechanism changes before reaching the 0 V cutoff, switching from intercalation to an additional minor pore-filling contribution, and thus a three-region mechanism (adsorption–intercalation–pore filling) was put forward. Alvin et al.,^41^ utilizing XRD, TEM, and GITT experiments, came to the same conclusions. However, NMR experiments have also revealed partial pore filling at intermediate voltages (0.2–0.1 V). Thus, a four-region mechanism was introduced (adsorption–pore filling–intercalation–pore filling).

### Scope of This Research Work

Aiming to understand the sodiation in HC systems, we perform a systematic density functional theory (DFT) study combined with electrochemical experiments. The model systems approach the structural configurations encountered in HC structures, covering the possible Na–HC interactions. We predict the relative stability of the phases by calculating the formation enthalpies and constructing the convex hull, the tie-line connecting the most stable configurations of each concentration. In this way, we obtain the energetically most favorable reaction pathway for the sodiation of HC models. This approach is extended to both Li and K exhibiting similar voltage profiles^13^ and placed in the perspective of recent literature. This brings forward a consistent and generalized storage mechanism of alkali metals in hard carbons, explaining capacity limitations as well as providing guidelines for optimizing HC anodes for alkali-metal battery applications.

## Methods

### Computations

Calculations based on the density functional theory (DFT) method were performed. The plane-wave Vienna Ab initio Simulation Package (VASP)^42^ was employed using the Perdew–Burke–Ernzerhof (PBE)^43,44^ exchange–correlation functional and the projector-augmented wave approach (PAW)^45^ to probe valence-core interactions. Van der Waals interactions were taken into account by applying the DFT-D3 corrections, while various results were additionally obtained with the DFT-D2 and DFT-D3(BJ) corrections or no corrections (PBE) for comparison. All results that do not specify otherwise are reported with the DFT-D3 corrections. The per-atom energy and force relaxation convergence criteria were set to 10^–4^ eV and 10^–3^ eV/Å, respectively. Spin-polarized calculations, commonly utilized in defective graphene,^39,46−50^ were tested and found to affect the energetics, and thus the calculated voltages, of configurations containing edge-surface environments while marginally affecting the rest of the configurations (Supporting Information A). Utilizing hundreds of DFT calculations covering a broad range of environments, we chose a balanced scheme where the bulk of the calculations (convex hull relaxations) does not include spin. The most stable structures were reoptimized in a subsequent step with spin polarization. Thus, all calculated voltages presented throughout this work include spin polarization.

The carbon-based model systems differ in size (ranging from 45 to 300 atoms) and shape (from simple graphene sheets to curved nanopores). The energy cutoff and *k*-point mesh were set to 500 eV and 7 × 7 × 1, respectively, for slab-based calculations (adsorption and intercalation). The slab vacuum was initiated at 20 Å, and extra vacuum space was introduced for each additional layer (C/Na/Li/K). The cutoff and *k*-point parameters were reduced to 400 and 3 × 3 × 1 (or 3 × 3 × 3 for nonslab configurations) for the larger models. A detailed overview and discussion of each model system are given in Supporting Information A.

The adsorption energy (*E*_ad_) of a number (*n*) of alkali-metal atoms on a carbon host is, herein, defined as
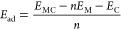
with *E*_MC_, *E*_C_, and *E*_M_ the total energies of alkali metal on carbon host, carbon host, and alkali-metal atom in the bulk crystal, respectively. The thermodynamic stability of the electrochemical systems was determined by calculating the enthalpies of formation (*E*_h_) according to the following equation^51,52^
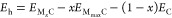
with *E*_M_max_C_ and *x* the maximum fraction of alkali metal in carbon host and the normalized concentration, respectively. Moreover, the average electrochemical potential between two phases with *x* and *y* alkali-metal concentrations can be obtained according to^53,54^
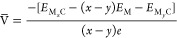


### Material Synthesis and Electrochemical Testing

The commercial hard carbon (CHC) sample used in the experimental part of this work was purchased from Guangdong Canrd New Energy Technology Co., Ltd. The CHC electrode was prepared by compressing active materials and sodium alginate at a weight ratio of 9:1 in deionized water solvent and casting the slurry on Cu foil, followed by a drying processing at 120 °C under vacuum for 6 h. The mass loading of CHC was controlled between 4 and 5 mg/cm^2^. CR2032 coin-type cells were assembled in an argon-filled glove box to conduct the electrochemical tests of CHC. Pure lithium/sodium/potassium foil, a solution of 1 M (mol/L) LiPF_6_/NaPF_6_//KPF_6_ in ethyl carbonate (EC) and diethyl carbonate (DEC) (1:1 in volume), and glass fiber (Whatman GF/D) were utilized as the counter electrode, electrolyte, and separator, respectively. The galvanostatic discharge and charge tests were performed on a Land BT3001A battery test system (Wuhan, China), cycled at the current density of 15 mA/g.

## Results

Our results are separated into smaller sections covering the possible interactions between alkali metals and HC. We initiate our investigation by revisiting the interaction of Na with the sp^2^ hybridization environment of a graphene sheet. Further, we evaluate the sodiation of an isolated graphene sheet (pristine, vacancy, and highly defective), bringing forward the redox potential and obtainable capacity. We continue by simulating various pore environments and models that combine several reaction mechanisms and conclude by simulating intercalation in graphene-based bilayers.

### Surface Adsorption

**Figure 2:**
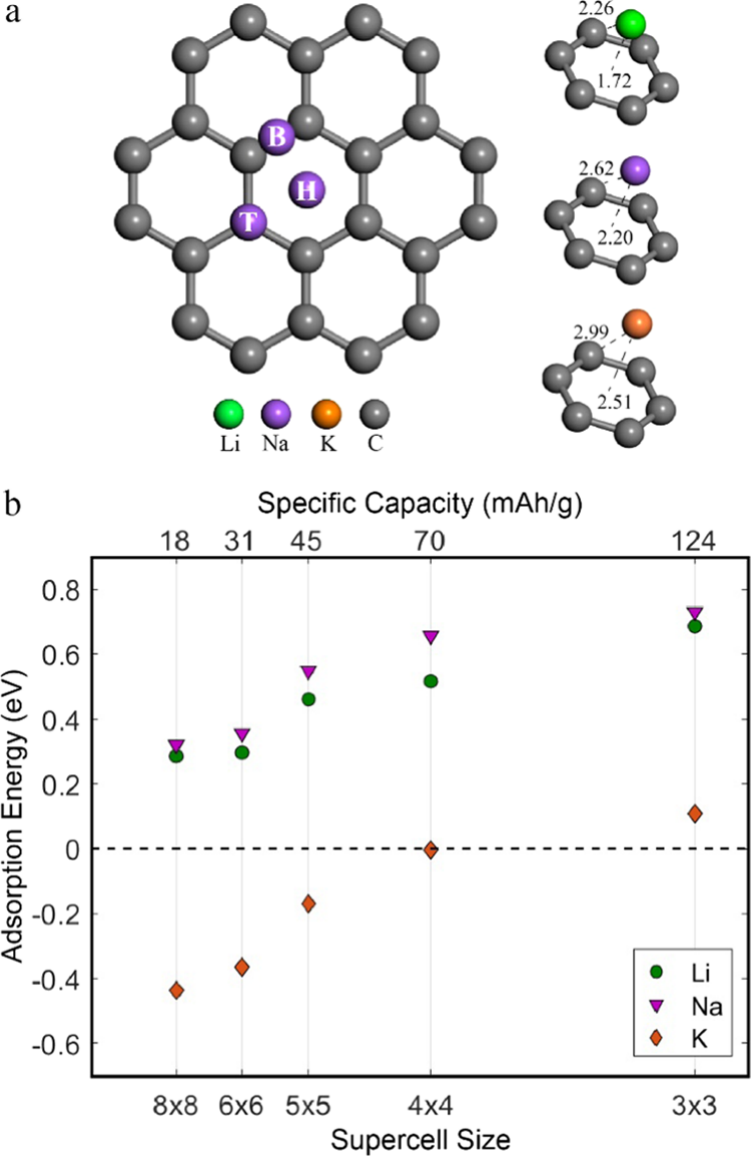
(a) (Right) Depiction of the hexagonal (H), bridge (B), and top (T) adsorption sites on a zoomed-in area of a 5 × 5 graphene supercell. (Left) Optimized configurations (distances noted in Å) of single-atom adsorption (Li/Na/K) on graphene. (b) Adsorption energies (eV) of Li, Na, and K on different pristine graphene supercells.

Single-atom adsorption on an isolated pristine graphene sheet is investigated on three different sites, namely, the hexagonal (H), bridge (B), and top (T), as indicated in Figure 2a. The hexagonal site, where six nearest carbon neighbors coordinate Li/Na/K, is more favorable than the bridge and top sites, consistent with previous reports.^55−63^ The Li, Na, and K atoms reside at a distance of 1.72, 2.20, and 2.51 Å above the graphene layer, obtaining Li/Na/K–C distances of 2.26, 2.62, and 2.99 Å, respectively.

The interaction strength between the Alkali metals and graphene can be quantified by calculating the adsorption energy. Calculations for a variety of graphene supercells, responding to different levels of surface coverage and thus different specific capacities, were performed (Figure 2b). According to the formulation we adopt herein, negative energies signify favorable adsorption on the graphene surface. The calculated energies are referenced to the alkali metal (energy of one atom in the metal crystal structure), providing direct insight into the measured voltage (at 0 K) in an alkali metal-graphene half-cell.

Li and Na result in positive adsorption energies, reflecting that the interaction with the sp^2^ graphene environment is unfavorable for adsorption. However, K achieves negative adsorption energies, indicating favorable binding (referenced to K metal) on the graphene’s surface in the dilute limit. All three alkali metals exhibit a similar trend; as the adsorbent concentration increases (by decreasing the graphene supercell size), the adsorption energy becomes considerably more positive (less stable).

Before moving toward more complex systems, it is crucial to address the accuracy of the DFT predictions based on the computational method. The Na results obtained herein are compared with the literature for various dispersion inclusion methods described in Supporting Information B. All techniques are qualitatively consistent, showing similar trends. However, we observe significant quantitative variations based on each study’s computational details (see, for example, the effect of different van der Waals corrections of Liang et al.^64^ in Figure S2). This observation is alarming considering the marginal differences the scientific community aims to probe near the plateau region (25–100 mV). Nonetheless, as we find in the latter part of this work, comparing the competing mechanisms for all three alkali metals relative to each other and with experiments will provide the necessary insights regarding their relative configurational stability and thus associated voltages.

### Mono/Multilayer Deposition and Plating

**Figure 3:**
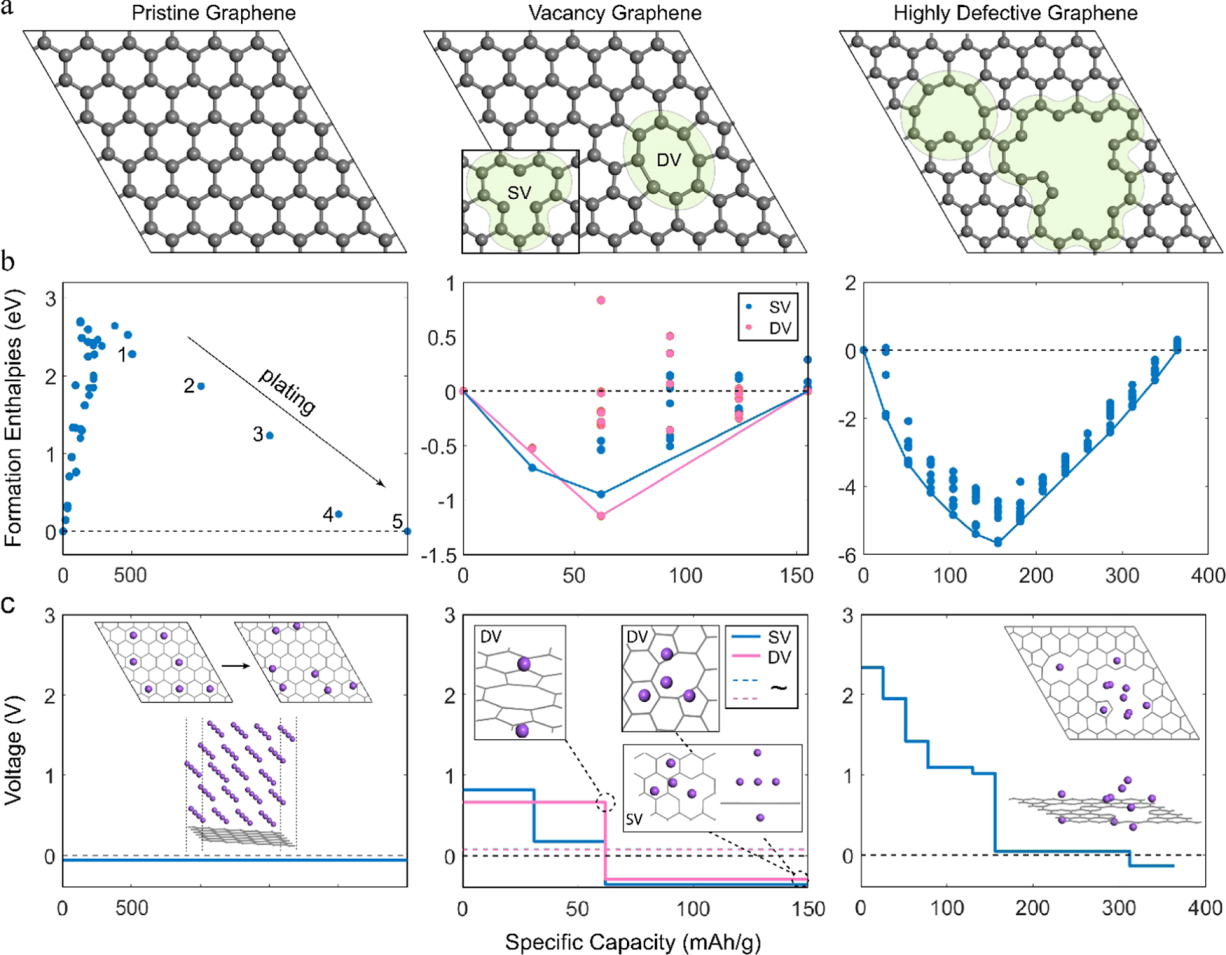
(a) (Left to right) Pristine, vacancy, and highly defective graphene configurations. (b) (Left to right) Formation enthalpies of Na configurations on top of pristine, vacancy, and highly defective graphene. The indicated numbers represent the number of (110) Na layers plated on the graphene surface. (c) (From left to right) Sodiation voltage profiles of pristine, vacancy, and highly defective graphene. The pristine graphene inset configurations demonstrate (top) clustering of Na atoms upon relaxation and (bottom) plated graphene with five (110) Na layers. The vacancy graphene inset configurations demonstrate (top) stable (positive voltage) single Na coverage of the double vacancy on both sides of the graphene sheet and unstable (negative voltage) four-Na atom island formation, and (bottom) unstable four-Na atom (or 5-atom for both sides) island formation on top of a single vacancy. The highly defective graphene inset demonstrates the stable Na graphene configuration at the end of the positive voltage plateau.

So far, we have established that the pristine graphene planes do not accommodate Na and Li at positive potentials. Nevertheless, it is instructive to evaluate the behavior of a graphene sheet under higher Na uptake as it will provide more insights into how Na interacts with the carbon environment. Subsequently, we explore the conditions (vacancies, discontinuities) that facilitate Na storage at positive potentials with respect to Na metal. Na was placed in different configurations (both two-dimensional, 2D and three-dimensional, 3D) upon three graphene types: pristine, vacancy, and highly defective (Figure 3a). Convex hulls were constructed by evaluating the formation enthalpies with respect to the reference phases (Figure 3b). The respective calculated voltage profiles are presented in Figure 3c.

As expected, for defect-free graphene surfaces, all intermediate configurations obtain positive enthalpies of formation, making the fully sodiated endmember, in this case, a plated Na multilayer, the most stable configuration. As a result, a first-order phase transition is predicted, where layered Na is plated on graphene at a constant potential (−0.05 V). Upon geometry optimization, Na clustering on the graphene surface is observed, indicating that the Na–Na interaction is the one to determine the configurational geometry over the lesser Na–C interaction. This observation is best demonstrated in the inset of Figure 3c, where Na is placed on the bi-adjacent hexagonal positions (adsorption-like placement), and upon relaxation, the atoms rearrange, obtaining closer distances (∼3.7 Å). Similar behavior is observed upon deposition of Na monolayers arranged in (100) and (111) surface geometries, as Na–Na distances adjust from approximately 4.1–3.7 Å. These distances resemble the (110) surface’s interatomic distances, which provide the densest Na monolayer with a surface coverage that responds to 496 mAh/g.

Moreover, the observed Na clustering spontaneously breaks the 2D symmetry in several geometry optimizations, revealing a preference for 3D clusters consistent with previous predictions.^29^ The plating process was simulated by adding four additional (110) Na layers on top of the most favorable monolayer. For each added layer, the voltage increased, converging to the plating potential, herein calculated at −0.05 V for five plated layers.

Next, we explore how graphene defects can affect the above picture. This approach is essential as DFT calculations have revealed that vacancies prefer forming in the free carbon surface of HC.^65^ It has been suggested that island formation (of a four or ten-atom Na cluster) in the vicinity of vacancies is thermodynamically stable and provides nucleation spots for Na clustering.^31,64^ Herein, we investigate the formation enthalpies of the four-atom Na island cluster, which is calculated as the most stable,^31^ on top of a single (SV) and double (DV) vacancy graphene sheet (3% vacancies). The calculations reveal that the formation of the Na cluster occurs in two stages. First, Na is introduced on the vacancies at high potentials belonging to the high-voltage sloping region. This occurs either at one or both (if accessible) sides of the graphene sheet. Once the dangling bonds of the vacancies are occupied, a first-order phase transition occurs, forming the favorable pyramid structure. However, this formation is predicted to be unstable vs Na metal as it obtains negative voltages. This discrepancy with literature might be because the cluster’s stability is often calculated as the average formation energy of the whole four-atom, or five-atom if both graphene sides are accessible, cluster. On the contrary, as presented here, a more detailed look following the lowest enthalpy path reveals a two-stage sodiation sequence. Ignoring this sequence and directly calculating the island’s formation average voltage results in stable configurations (dotted lines at slightly positive potentials in Figure 3c). Results in Figure 3c include Na dissipation on both sides of the vacancy graphene sheet. Redrawing the convex hull and voltage profile while ignoring the sodiation of the opposite graphene side does not change the observed trend.

A highly defective graphene sheet was tested as well. This sheet was inspired by the highly defective structure proposed by Shinn et al.,^66^ excluding the variety of heteroatoms to simplify the calculations. The defective environment presents wide gaps of carbon discontinuities (∼5 Å) and, thus, various Na binding spots. The sodiation initiates a solid-solution reaction (sloping region) at the inner walls of the discontinuity and other vacancies, transitioning to a first-order phase reaction of an island formation directly on top of the discontinuity. Unlike those predicted on top of isolated vacancies, these islands are thermodynamically favorable, obtaining a slightly positive potential in the plateau region. The island consists of eight atoms being 3.57 Å apart on average. Further, expanding this island or sodiating the rest of the graphene sheet results in negative potentials, similar to the pristine graphene case.

### Defect Adsorption

**Figure 4:**
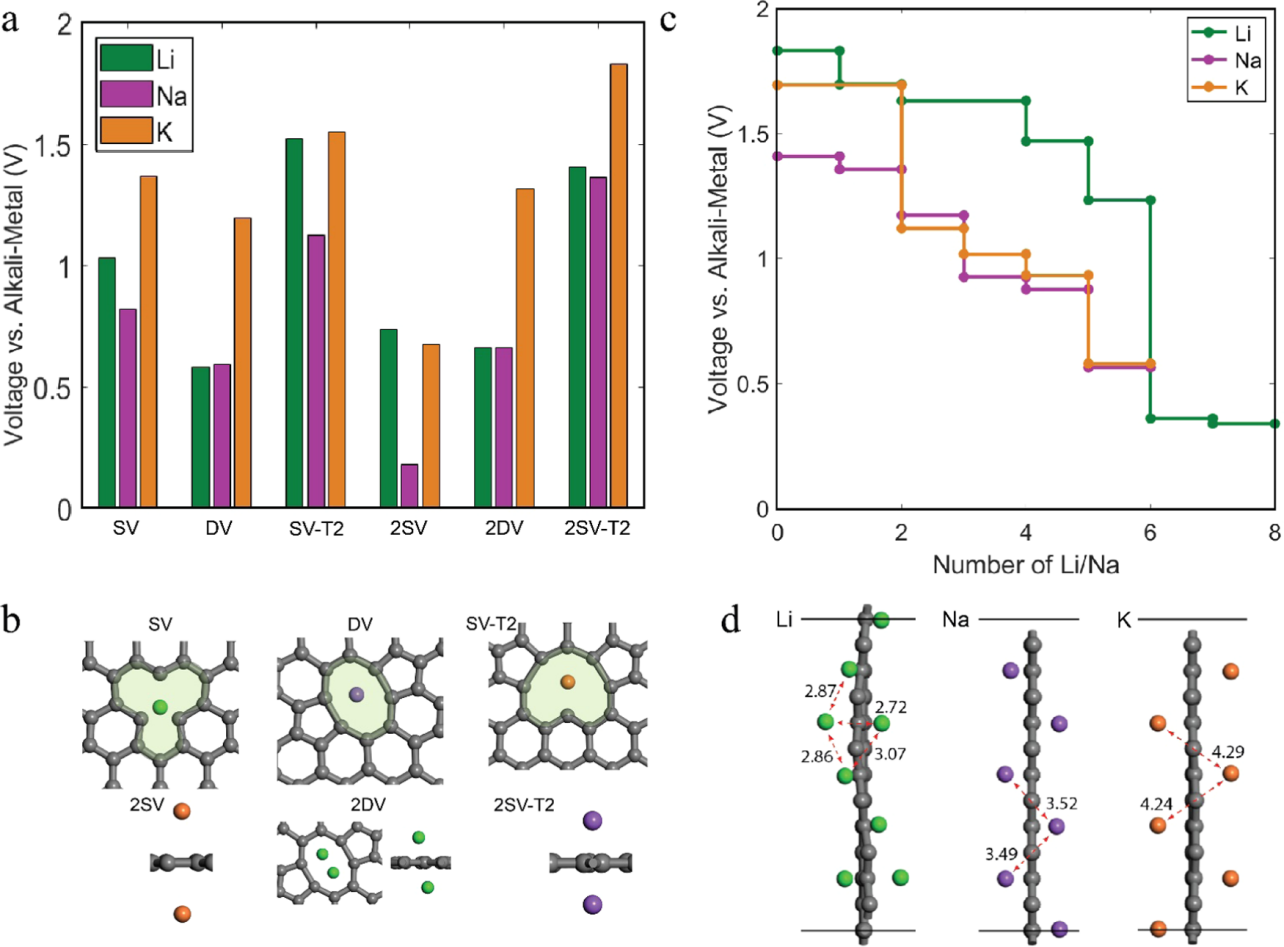
(a) Voltage of alkali metal storage in various defect environments. The number “2” in front of the vacancy notation indicates storage on both sides of the graphene sheet. (b) Configurational environment of alkali metal storage on top of vacancies. (c) Voltage of alkali metal edge adsorption. (d) Configurational environment of alkali metal edge storage.

It is evident from the above discussion that defects (vacancies and edges) are essential to enable Li and Na storage in hard carbon. Vacancy and edge-surface adsorption results for Li, Na, and K are presented in Figure 4. In Figure 4a,b, we observe several defect environments with broad adsorption voltage distributions belonging to the high-voltage sloping region. Depending on the choice of the graphene supercell, the vacancy concentration percentage affects the calculated result as a denser vacancy environment leads to a denser alkali metal distribution, lowering the voltage. This effect is extensively demonstrated by Datta et al.,^67^ who studied Na adsorption on defective graphene sheets containing up to 100% (double and Stone–Walls) vacancies. The sodiation voltage initiates at higher potentials and gradually decreases to 0 V in a sloping voltage curve. A sloping voltage profile at positive potentials is also predicted for edge-surface adsorption (Figure 4c,d), resembling a solid solution edge-surface filling. The voltage slopes span from positive (∼2 V) and gradually decrease toward negative potentials with increasing alkali-metal concentration. All three alkali ions utilize the same binding locations, the exposed carbon edges; however, the configurations’ specific geometry depends upon the size of each alkali metal. On average, Li–C, Na–C, and K–C distances are 1.98, 2.37, and 4.26 Å, respectively. Li adsorption allows more dense rhombic-like configurations (Li–Li distance of 2.72 Å). Nevertheless, this configuration is unfavorable for Na and K, which only obtain a stable zigzag configuration.

In retrospect, HC vacancies and edges offer binding spots that contribute to the sloping region’s obtainable capacity and could potentially provide a favorable nucleation spot for Li, Na, and K clustering; however, island formation requires a highly defective environment and not isolated vacancies, as demonstrated in Figure 3, to be stabilized.

### Pore Filling

So far, we have considered a single graphene sheet for our investigation. Next, we will introduce a second pristine graphene layer to our system, evaluating how it affects storage capabilities. This approach simulates the pore-filling mechanism as the Na-mono/multilayers are “sandwiched” between graphene sheets. The reader should not confuse this process with intercalation as Na exists in a denser form than the NaC_8_ and NaC_6_ phases, and the graphene sheets are separated by at least 6.5 Å, which is the optimized distance of “sandwiching” a (110) Na monolayer.

**Figure 5:**
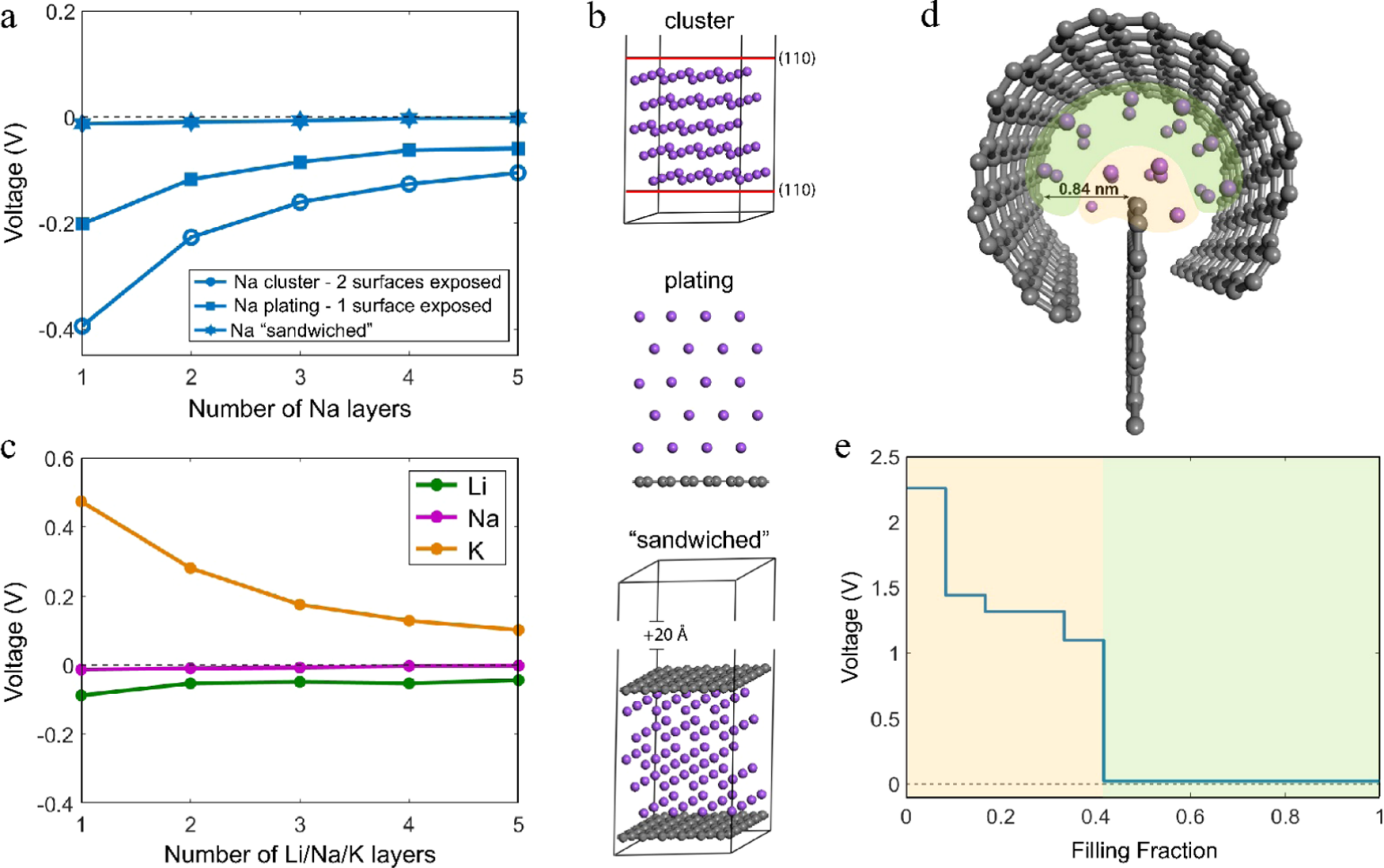
(a) Voltage profile of one to five Na layers in cluster, plated, and sandwiched configurations. (b) Depiction of the cluster, plating, and sandwiched configurations. (c) Voltage profile of sandwiched Li, Na, and K layers. (d) Depiction of the fully sodiated “power-button” configuration. (e) Sodiation voltage profile of the power-button configuration.

Figure 5a presents the voltage evolution of isolated (one to five) (110) Na layers, compared to plating and sandwiching these layers on and within pristine graphene, respectively. In this way, we can determine the contact properties between graphene and layered Na and the effect of this contact on the voltage. As discussed in the previous section, the (110) surface is selected as the most favorable monolayer on graphene, naturally forming upon relaxation.

The Na layers (Figure 5b) present two exposed (110) surfaces, introducing an energy penalty per exposed surface that brings the voltage well below 0 V vs Na metal. The voltage rises for each surface contact with pristine graphene introduced, indicating that the contact improves the thermodynamic stability of the system. Carbon cancels the energy penalty of the exposed surfaces replacing it with lower interface energy, lowering the configurations’ energy and thus enhancing phase stability. As a result, we expect that Na clusters in the HC environment will try to minimize their exposed surfaces by being enclosed or wrapped up by carbon sheets in a manner that will favor pore-filling. Additionally, this is the first indication that a completely filled pore is preferred to a partially filled one, as the latter will pose exposed surfaces. The voltage of the sandwiched Na roughly is predicted to be close to 0 V; however, the absolute voltage value might be underestimated and greatly depends on the computational method. It also depends on the reference host, in this case, infinite parallel perfect graphene sheets.

The same thorough evaluation was performed for Li and K. In Figure 5c, we present the voltage profiles of the sandwiched Li, Na, and K layers. Li exhibits a similar trend at slightly negative voltages, lower than that of Na (∼45 mV). The positive and gradually decreasing voltage for K, on the other hand, is attributed to the favorable surface adsorption predicted in the previous chapter, explaining why a sandwiched K monolayer is more favorable than the multilayers. The relative positions of the voltage lines for Li, Na, and K (Figure 5c) are determined by two factors: the surface energy penalty and the stability of the surface–carbon interface contact. Calculations on the (110) Li, Na, and K surfaces are performed and presented in Supporting Information C, matching the experimental results and previous computational studies. The energy for forming the (110) surface increases accordingly, *E*_K_ < *E*_Na_ < *E*_Li_. In addition, forming the sandwiched configuration reveals that the surface–carbon contact favors the alkali metals in the same order, rationalizing the observed voltage trend. We expect that the observed voltage differences between the alkali metals will exhibit themselves in the respective pore-filling mechanisms.

Before examining the above proposition further, we gain additional insights into the nature of the pore-filling reaction mechanism, including a possible nucleation center. Figure 5d,e demonstrates a power-button configuration and the sodiation voltage profile. We observe that sodiation initially accumulates on the edges, producing a sloping voltage curve with increasing Na concentration. The edge surface forms the nucleation center, as subsequently, the pore is filled (Na top layer in contact with the curved graphene sheet), resulting in a constant positive potential, producing the low-voltage region, indicating that this is responsible for the experimentally observed Na–HC’s plateau. In Supporting Information D, calculations on a vertical “edge to plane” configuration are depicted where this phenomenon is presented again with exaggerated sodiation steps, showing both the effect of the nucleation center and the preference of a completely filled pore compared to a partially filled one.

Finally, joining all storage phenomena within one morphology, a wedge-pore superstructure of semiparallel layers (Figure S1e) is considered, including edge surfaces, vacancy defects, and pores, to approximate the actual situation of HC. The pore size is 3.70 × 0.74 nm, and the interlayer sheet distance ranges from 0.56 to 1.14 nm, a range that includes the computationally predicted sheet distances for optimally sandwiching one, two, and three Na layers.

**Figure 6:**
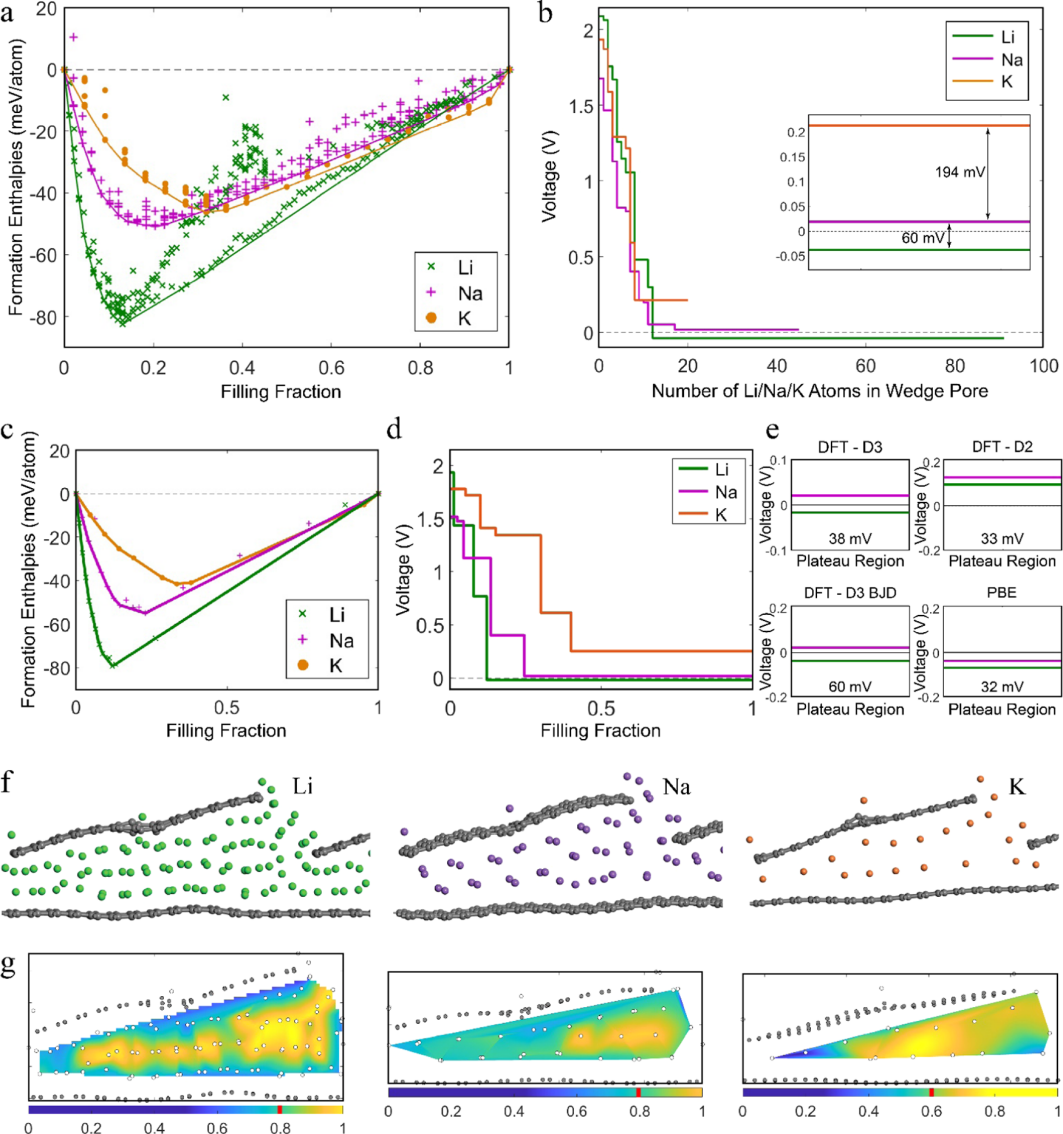
(a) Convex hull of Li, Na, and K into the wedge pore (frozen carbon matrix). (b) Voltage profiles of Li, Na, and K into the wedge pore (frozen carbon matrix). (c) Convex hull of Li, Na, and K into the wedge pore (fully relaxed). (d) Voltage profile of Li, Na, and K into the wedge pore (fully relaxed). (e) Li vs Na plateau with different computational methods. The number indicates the Na–Li voltage difference. (f) Li, Na, and K configurations of the completely filled pore. (g) Cross section of the pore’s electronic status for the completely filled configuration. Values close to 1 indicate metallic behavior as the valence charge resides with the alkali atom. Values close to 0 indicate ionic behavior as the valence charge is donated to the carbon matrix. The red stripe in the color bar specifies the average value. Note the different color scaling for K.

The pore is gradually filled with alkali metals in different competing configurations. Initially, the carbon matrix is kept frozen for the alkali metals to relax (Figure 6a). Subsequently, the lowest enthalpy points were fully relaxed (Figure 6c), allowing the carbon matrix to adjust and wrap around the alkali metal atoms. Based on the lowest enthalpy paths (Figure 6a,c), the voltage profiles are calculated and presented in (Figure 6b,d) for the frozen and fully relaxed systems.

Lithiation, sodiation, and potassiation initiate on the edge surfaces, consistent with the previously presented analysis. Subsequently, upon increasing the alkali-metal concentration on the edges-surfaces, defect (vacancy) adsorption becomes competitive and eventually takes over as being energetically more favorable at higher concentrations. The sloping voltage profile concludes at which stage the remaining positions on the edge surfaces are filled.

Additional sodiation initiates the pore-filling stage. Configurations representing partially filled pores in the convex hull are unstable compared to the combination of empty and completely sodiated endmembers, consistent with our results presented earlier. This suggests that pores are filled one by one, keeping the change in free energy constant, and as a result, the voltage profile transitions from a sloping curve to a constant voltage plateau, successfully capturing the experimentally observed behavior. Compared to the completely filled pore endmember, the instability of the intermediate phases indicates that the exposed surface (when Li/Na/K does not wet the carbon pore walls) introduces an energy penalty. The optimized configurations of the filled pores are presented in Figure 6f. The average Li–Li, Na–Na, and K–K distances are 2.84 and 3.57, and 4.76 Å, respectively.

We observe that the relative voltage difference between the three alkali metals (Figure 6b,d) reflects our earlier conclusions on the sandwiched pore (Figure 5c). K appears at significantly higher voltages than Na, exhibiting the voltage plateau at positive voltages. The Na plateau occurs at slightly positive voltages matching the experimentally observed behavior. On the other hand, the Li plateau stabilizes at negative voltages, indicating that no obtainable capacity related to pore-filling will be delivered in a Li-HC battery.

As discussed in the first section of this work, we need to be critical on the accuracy of DFT, especially when probing differences in the range of a few millivolts. For this reason, we repeated the plateau voltage calculation with different computational methods (Figure 6e). We observe that the absolute position of the voltage lines slightly swifts; however, the trend and relative stability of the Li and Na plateau lines remained consistent for all methods, and in all cases, Li falls short of the Na line by, on average, 40 mV. Moreover, the Li pore-filling plateau is calculated at a higher (less negative) voltage than the Li plating potential, so we expect it to occur before reaching the characteristic V shape of Li nucleation and plating.

Bader charge analysis on the electronic state of the completely lithiated/sodiated/potassiated pore is presented in Figure 6g. The calculations suggest a semimetallic state with an average electron transfer from Li and Na to the carbon matrix of 20%. The Li layers in contact with the carbon walls (interface) have a somewhat higher electron transfer than the Na ones. However, an extra intermediate Li layer can fit in the same pore size so that the average Li pore-filling picture appears the same as Na’s. The electronic analysis results are consistent with experimental NMR experiments^28,30^ revealing semimetallic Na clustering and DFT-machine learning calculations^32^ depicting partial electron transfer in the same range. *K* donates 40% of its valence charge to the carbon matrix.

### Experimental View

Let us now put our results into perspective by comparing them with experimental observations. Kubota et al.^13^ performed a thorough structural analysis of HC samples annealed at different temperatures, correlating the electrochemical behavior of Li, Na, and K with structural characteristics. These findings suggest that higher annealing temperatures induce fewer defects, dangling bonds, surface area, and larger pore sizes. Following the electrochemical behavior of Li, Na, and K for increasing annealing temperatures, a gradual decrease in the available capacity of the sloping region is observed. This is consistent with the adsorption mechanism, as fewer available surfaces and defects present fewer binding spots for the incoming alkali metals.

**Figure 7:**
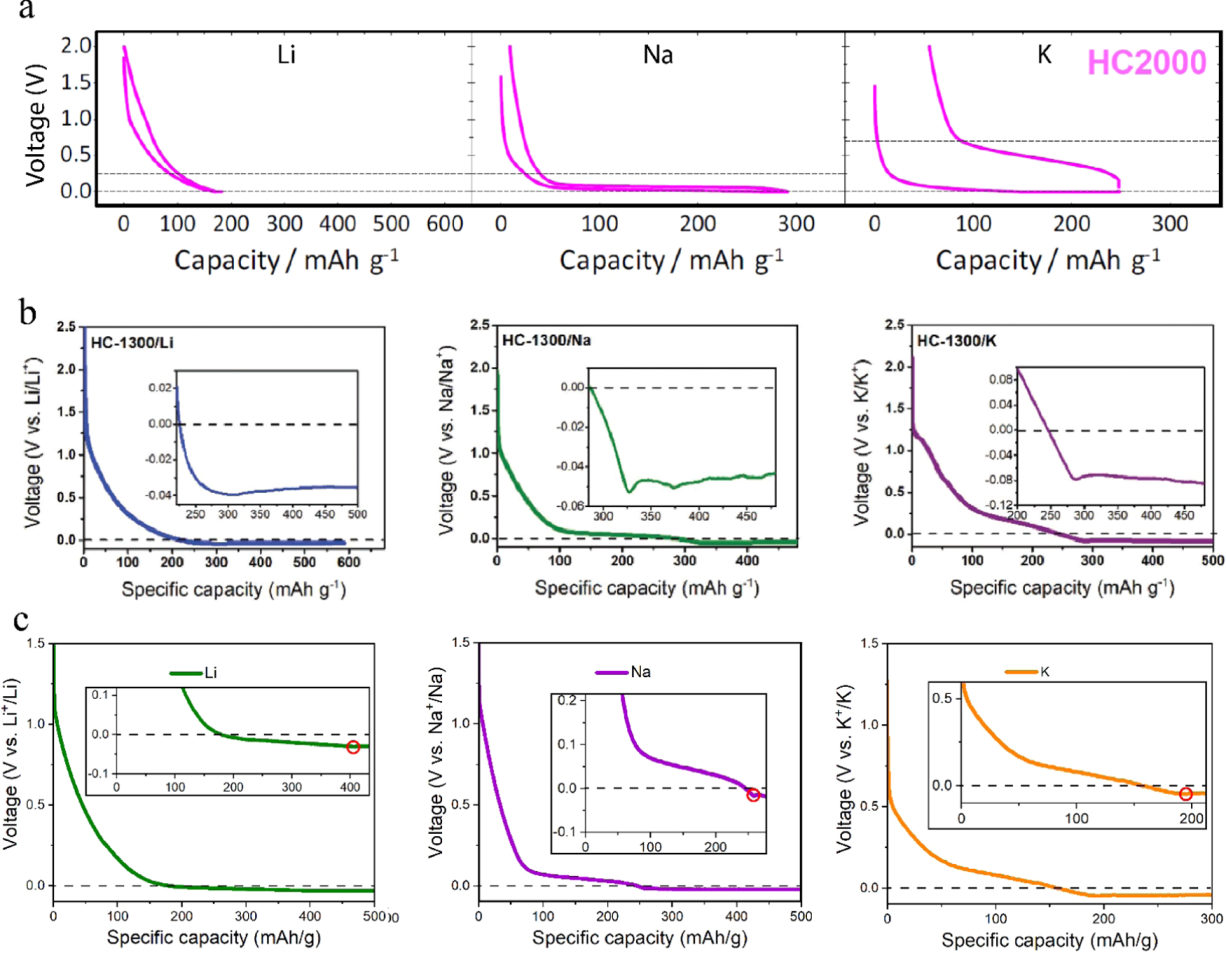
(a) Galvanostatic discharge of Li, Na, and K in HC annealed at high temperatures with the cutoff voltage at 0 V. Reproduced from Kubota et al., Chem. Mater. 2020, 32, 2961–2977. Copyright 2020 American Chemical Society. (b) Discharge profiles of HC-1300/Li, HC-1300/Na, and HC-1300/K collected when the voltage decreased below 0 V. Reprinted with permission from Alvin et al., Adv. Energy Mater. 2020, 10, 2000283. Copyright 2020 Wiley. (c) Galvanostatic discharge curves in Li, Na, and K half-cells, respectively. The red circle indicates reaching the V shape.

Further, for increasing annealing temperatures, a larger plateau region evolves for Na and K, consistent with the pore-filling mechanism as the number and size of pores increase. At first glance, the evolution of the plateau region with increasing temperature looks different for Li as the voltage line hits the 0 V cutoff before exhibiting a voltage plateau at positive potentials. To demonstrate that, we isolated the electrochemical results (for the higher annealing temperatures) of Kubota et al.,^31^ presented in Figure 7a. However, experiments^36^ that include overdischarging below 0 V (Figure 7b) reveal that the Li voltage plateau exists a few mV under the Na voltage line at slightly negative potentials, matching the DFT calculated behavior for the pore-filling mechanism.

To further investigate the above scenario, a commercially available HC electrode (CHC) was prepared for Li and Na, and K half-cells, and overdischarging below 0 V was applied. The goal is to reproduce the voltage features observed in the literature, show the hidden Li plateau below 0 V, and measure the plateau voltage difference. Our experimental results are presented in Figure 7c and Supporting Information E, showing that the plateau capacity for Li below 0 V vs Li/Li^+^ (228 mAh/g) is much higher than that of the Na case (13 mAh/g). In other words, for Li, the long plateau before reaching the V shape, appears below 0 V. In contrast, the Na plateau appears at positive potentials reaching the V shape immediately after dropping below 0 V. Finally, the K plateau appears more gradual and at higher potentials from the Li and Na counterparts. This behavior matches the computational pore-filling description presented in the previous section.

As a result, Li, Na, and K follow the same voltage trend, initializing with adsorption on edges and defects followed by first-order phase transition of alkali metal insertion into nanopores. The specific position of the voltage plateau for each alkali metal depends on the energy specifics of surface and carbon–metal interface thermodynamics. If the closed pores are too large, the graphene sheets of the wedge-pore are too far apart, and the exposed surface will likely drop the voltage below the 0 V cutoff before the first-order phase transition manages to wet the whole surface, limiting the offered capacity. Thus, the competition between nucleation and surface coverage suggests that an optimum pore size should exist. For Na, the optimum pore size is shown to be about 1 nm both experimentally^13^ (through a maximum in the observed capacity with increasing pore size) and computationally.^31^ The pore size distribution and especially pore shape significantly determine the storage process and can be used to design and optimize hard carbon materials’ performance.

### Intercalation

**Figure 8:**
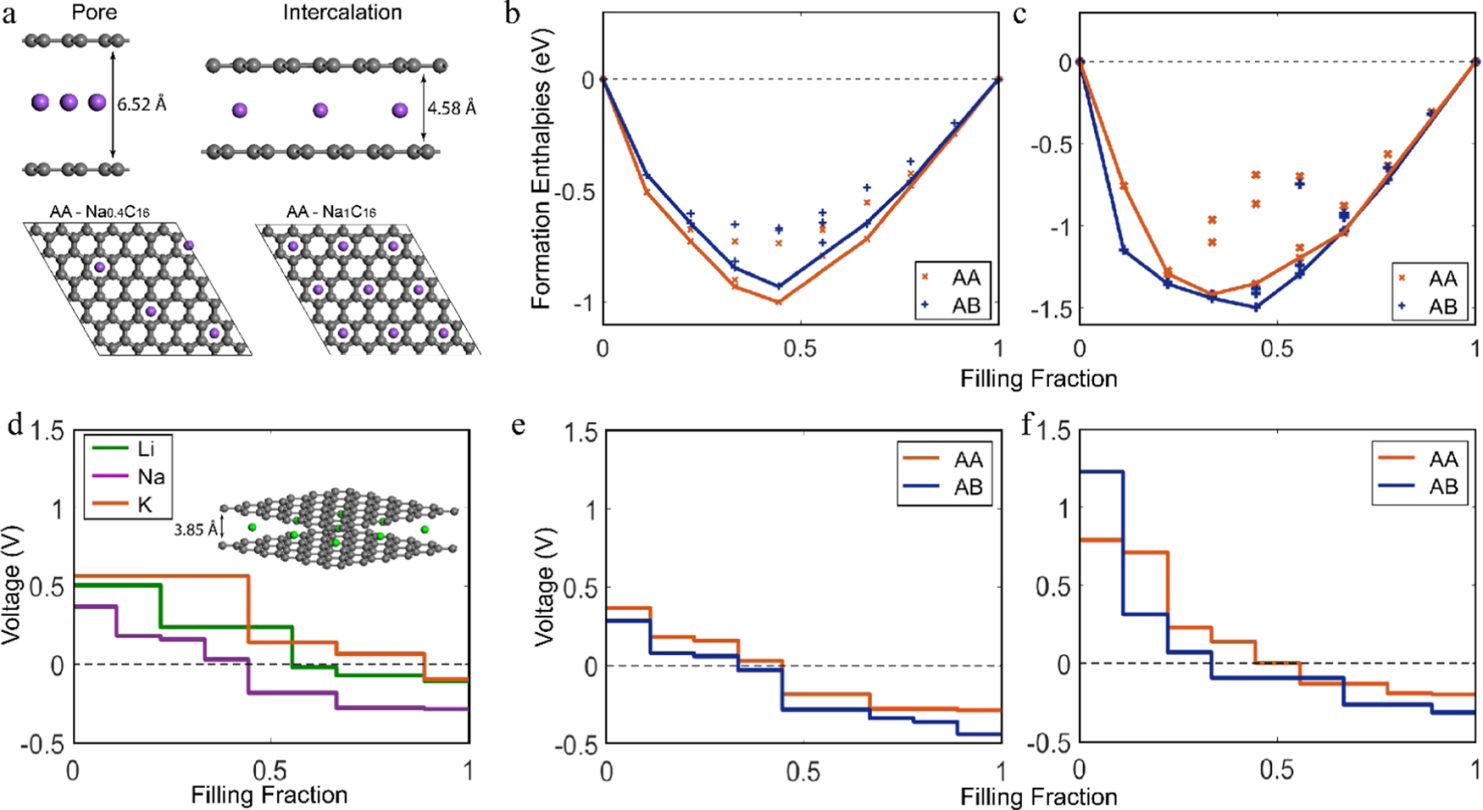
(a) (Top) Visual representation of pore vs intercalation configurations. (Bottom) Depiction of Na_0.4_C_16_ and Na_1_C_16_ intercalated phases. (b) Formation enthalpies of Na intercalation in AA and AB pristine graphene bilayer. (c) Formation enthalpies of Na intercalation in AA and AB graphene bilayer containing a single vacancy. (d) Voltage profile of Li, Na, and K intercalation in AA pristine graphene. The inlet demonstrates the optimized, fully intercalated Li phase. (e) Voltage profile of Na intercalation in AA and AB pristine graphene bilayer. (f) Voltage profile of Na intercalation in AA and AB graphene bilayer containing a single vacancy.

The last part of our investigation focuses on intercalation. We differentiate intercalation from pore-filling as, in this section, Na is not clustered in (semi)-metallic formations but instead coordinated by the carbon environment of two adjacent graphene sheets (Figure 8a). Configurations of Na intercalated in a graphene bilayer slab were computationally optimized, covering the concentration range 0 < *x* < 1 in Na*_x_*C_16_ for both AA and AB graphene stackings. The relative phase stability of the intercalated phases was investigated by constructing the convex hull and following the tie-lie connecting the most stable phases. Na intercalates through a solid-solution process (Figure 8b), where most intermediate compositions are energetically accessible. This behavior contradicts the widespread assumption that possible intercalation of Na due to the larger interlayer distancing should lead to a first-order phase transition, producing a flat voltage profile in the low-voltage region,^21,34^ similar to Li intercalation into graphite.^63,64^

The resulting continuous decrease in voltage for Na intercalation in a pristine graphene bilayer quickly drops below 0 V, already when reaching the Na_0.4_C_16_ composition, covering an +0.37 to −0.29 V range (for the lower energy AA stacking) (Figure 8e). With increasing bilayer distance, the voltage slope generally increases, reaching a maximum at 4.48 Å for Na intercalation, which is predicted to be the optimum bilayer distance for the Na_1_C_16_ phase, in agreement with previous literature (4.52 Å).^39^

The formation voltages of the intercalated phases strongly depend on the graphene interlayer distance of both the pristine graphene bilayer as well as the Na intercalated graphene configurations. This behavior is investigated by calculating the voltage profiles for a wide range of interlayer distances ranging from 3.34 to 4.74 Å (Figure S5). For bilayer distances lower than 3.94 Å, intercalation was not possible at positive potentials, even for the very dilute phases.

The convex shape changes considering sodiation in a defective graphene bilayer, in this case, a graphene bilayer with a single vacancy (Figure 8c,f). The voltage initiates at higher potentials following a steeper slope reaching a tale that flattens out slightly, allowing the Na_0.66_C_16_ phase to be reached for high interlayer distances (4.48 Å) and AA stacking. However, the nature of the sodiation process remains the same, resembling a solid solution insertion.

Several DFT studies have indicated that intercalation is possible in the constant voltage plateau region, reaching the Na_1_C_16_ phase for certain interlayer distances (3.75–4 Å) and misalignment of the graphene sheets.^21,39,41^ However, ignoring the formation enthalpies and directly calculating the average voltage of the fully sodiated phase might be misleading. For example, ignoring the predicted voltage slope in our study, the average voltage of forming the Na_1_C_16_ phase is slightly positive, falsely appearing in the plateau region. Nevertheless, analyzing smaller compositional changes based on the convex hull reveals the true nature of a graphene bilayer sodiation.

Based on the above DFT analysis and the experimentally determined parameters of the HC structures (minimal stacking and interlayer distance up to 4 Å), only minor intercalation contributions are rationalized, contributing (more) in the sloping and (less) in the plateau regions. It would be interesting to investigate how the voltage shape changes upon introducing more vacancies. Perhaps in future work, an aim can be the inclusion of more convex hull constructions with highly defective graphene bilayers. In this way, we could explore whether the voltage flattens further, allowing some capacity contributions in the plateau region, forming the Na_1_C_16_ phase. However, this will be possible for greatly expanded graphene layers (4.48 Å). Flattening but still sloping voltage regions have been demonstrated with DFT in defective graphene bilayers in the literature^68−70^ with the calculated D-spacing range (3.7–4.6 Å), in agreement with the herein predicted optimized bilayer distances for intercalated Na. Li and K intercalation in a pristine graphene bilayer is also performed. The convex hull indicates a solid solution behavior similar to the Na case, leading to a sloping voltage curve presented in Figure 8d.

## Conclusions

**Figure 9:**
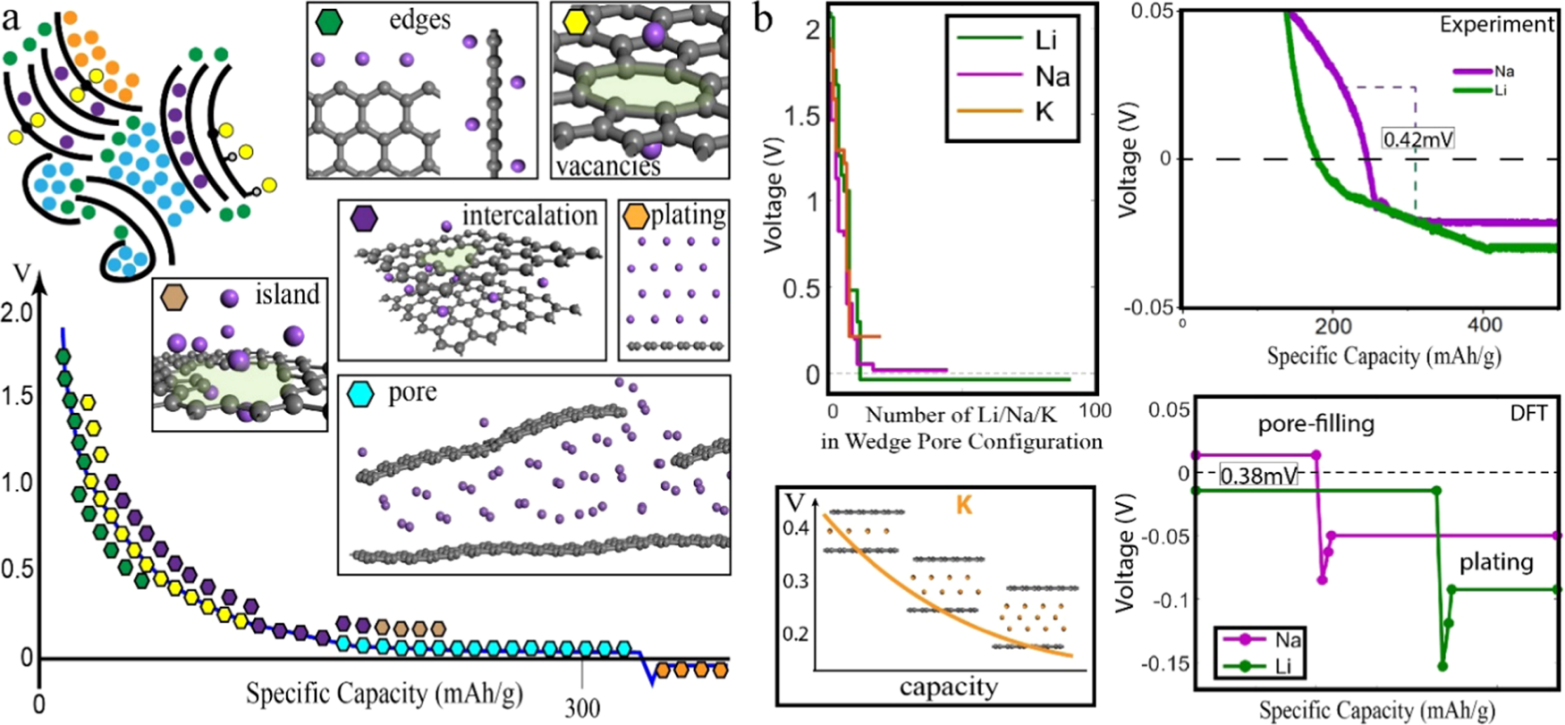
(a) Summary of the Na voltage profile and the respective mechanisms. (b) Summary of voltage-feature differences between the alkali metal voltage profiles. (Top left) Voltage vs number of alkali metals in the wedge pore configuration. (Bottom left) Indicative K behavior in a pore, pore capacity is gradual compared to Na and Li. (Top right) Zoom-in of the experimental cycling of Li and Na focusing on a narrow 0.1 V voltage window. The plot is squished in the x-axis to reflect both Li and Na’s plateau, V shapes, and plating potentials. The average plateau difference is 42 mV, where Na appears at positive and Li at negative potentials, respectively. (Bottom right) DFT picture of the end of discharge. The plateau difference is 38 mV, where Na appears at positive and Li at negative potentials, respectively. The V-shaped voltage construction between the calculated plating and pore-filling plateaus is indicative. The two intermediate points in the V shape are the potentials of three and four alkali metal layers on graphene.

We explore the nature of Li, Na, and K storage mechanisms in HC by evaluating the formation enthalpies in various model systems using DFT. Figure 9a summarizes all of the investigated mechanisms distributed on the Na voltage curve, rationalizing the observed behavior. The reaction initiates at edges, defects, and vacancies where alkali metals are adsorbed in a wide range of energies, giving rise to the initial part of the sloping voltage plateau. The sharp voltage drop is a function of alkali metal concentration, covering the free dangling bonds of the defective carbon environment. Further, intercalation in defective graphene bilayers takes over, where contrary to popular belief, the nature of the reaction does not change, representing a solid solution reaction. As a result, intercalation is part of the sloping voltage curve, with only minor contributions to the plateau region. However, the predictions for intercalation require large interlayer distances between the graphene sheets and do not allow all of the available capacity, as highly intercalated phases are thermodynamically restricted. Finally, the process concludes, initiating nanopore filling, where nanopores are filled one by one producing a flat voltage plateau.

The reaction pathway is similar for the investigated Li and K cases, with differences highlighted in Figure 9b. Even though the Li pore-filling process seems different at first glance, careful evaluation of the hidden capacity below the 0 V line establishes that the three alkali metals behave similarly, putting forward a unified reaction pathway. The energy penalty of exposed surfaces of alkali metal clusters combined with the stability of the metal–HC interface contact is responsible for the value of the respective voltage plateaus and determines the available pore-filling capacity in alkali metal–HC batteries. The calculated voltage profiles in all pore-filling systems (Figures 5c, 6b,d,e, and 9b) capture the experimentally determined voltage difference between the alkali metals (Figures 7 and 9b), showing Na’s plateau in slightly higher potentials of about ∼40 mV from Li’s plateau. In the K case, the pore-filling stage is predicted to evolve more gradually as the voltage of a sandwiched K monolayer appears higher than that of a larger K nanocluster. The voltage plateaus attributed to pore-filling appear computationally and experimentally at higher potentials than the “V” shape voltage feature, signifying the plating process

Since the sp^2^ hybridization plane is unfavorable for Li and Na bonding, the pore-filling mechanism requires key nucleation centers to occur. Isolated defects can only provide this environment for up to two Na layers as island formation is shown to be unstable. On the other hand, edges and island formation on top of discontinuities of highly defective graphene sheets can play that role. The above suggest that there should be an optimum pore size, which has been experimentally shown for Na, where we experience a maximum in the obtained capacity with increasing annealing temperature and thus pore size.^13^ An exciting direction for future research is investigating the effect of various electrolytes on the behaviors presented herein. Various examples in the literature^11^ have reported the co-intercalation of electrolyte molecules which are expected to alter the interaction potentials in the HC environments. Our findings offer an in-depth view of the reaction mechanism of alkali metals in HC. These insights enrich the fundamental understanding of HC-based electrodes and contribute to advancing the optimization of HC-based batteries.

## Supporting Information Available

Presentation and discussion of all hard carbon models, effects of spin-polarized calculations, comparison of Na adsorption with the literature, surface energy calculations, convex and voltage of edge to plane configuration, the first cycle of Galvanostatic discharge, and Na into a bilayer intercalation calculations (PDF)
