# Supplementary material for: Role of Defects, Pores, and Interfaces in Deciphering the Alkali Metal Storage Mechanism in Hard Carbon
Source: ACS Appl Energy Mater. 2022 Dec 29;6(1):127–40. doi: 10.1021/acsaem.2c02591 (PMC9832432; doi:10.1021/acsaem.2c02591)
Supplement: Supplementary file 1 — ae2c02591_si_001.pdf [file ae2c02591_si_001.pdf]

Supplementary Information for

The Role of Defects, Pores, and Interfaces on Deciphering the Alkali Metal Storage Mechanism in Hard Carbon.

Alexandros Vasileiadis[1]*, Yuqi Li[2,3], Yaxiang Lu[2], Yong-Sheng Hu[2,3], Marnix Wagemaker[1,]*

1. Storage of Electrochemical Energy, Department of Radiation Science and Technology, Faculty of Applied Sciences, Delft University of Technology, Mekelweg 15, 2929JB, Delft, The Netherlands

2 Key Laboratory for Renewable Energy, Beijing Key Laboratory for New Energy Materials and Devices, Beijing National Laboratory for Condensed Matter Physics, Institute of Physics, Chinese Academy of Sciences, Beijing 100190, China

3 College of Materials Science and Optoelectronic Technology, University of Chinese Academy of Sciences, Beijing 100049, China

*Corresponding author.

Email: a.vasileiadis@tudelft.nl, m.wagemaker@tudelft.nl

Supplementary Information A

Calculation Details

Configurations and Selection Criteria

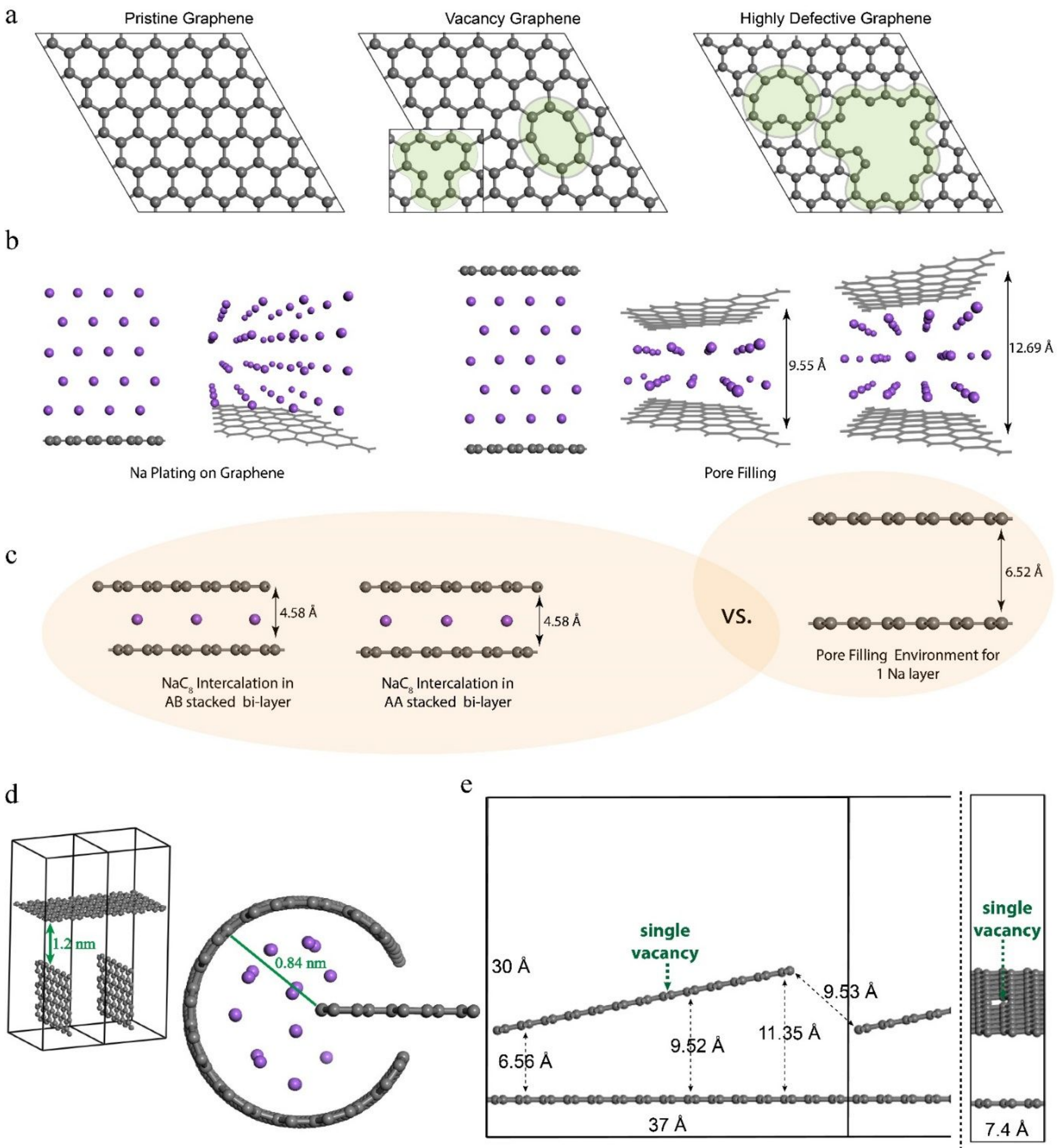

Figure S2 a) Pristine, vacancy, and highly defective graphene slab DFT models with 20 Å vacuum. b) (left) Plating DFT slab model with five alkali metal layers (right) "Sandwiched" DFT slab environments. The slab configuration was increased so that 20 Å vacuum remains. c) intercalation configurations vs. the pore configuration, the investigation was performed with and without vacancies. d) Vertical "edge to plane" and "Power button" configurations. e) Wedge pore configuration with semi-parallel sheets. The periodic boundary conditions suggest that a 20 Å vacuum separates two adjacent wedge pores in the z-direction, and the pore is infinite in the y-direction.

Hard carbon has a very complex configurational environment. Thus we aim to isolate the various environments in simple systems fitted for DFT ab-initio simulations. Each system aims at capturing a specific alkali metal–hard carbon interaction. Hard carbon contains several defective graphene sheets. Thus we begin the investigation by studying simple graphene sheets with different types of vacancies (FigS2 a). We additionally study the behavior of alkali metals upon pristine graphene for comparison. As indicated in the methods section for each slab configuration, we introduce at least a 2 nm vacuum. For the pristine graphene, several supercells were investigated, ranging from 3x3 to 8x8. The vacancy environments in Figures 3 and 4 are tested in supercells containing 72 C atoms, except for the highly defective sheet, which was tested in a supercell containing 96 C atoms. We also aim to investigate the behavior of alkali metals near edge environments. The 72 C atom supercell was placed parallel to the vacuum slab for these calculations to expose the edges to the vacuum. Each parallel sheet is at least 1.5 nm apart from the next sheet.

We investigate the fundamental interaction of alkali metal–hard carbon interfaces for the pore environment. For this purpose, the "sandwiched" configuration is beneficial. This configuration is built by introducing a second graphene layer of 72 C on top of the lithiated/sodiated/potentiated initial graphene sheet (FigS2 b). More vacuum is introduced with the addition of each layer to keep the vacuum distance. Further, we tested different pore environments with alkali metal accumulation centers ("vertical edge to plane," "power button," and "wedge pore" configurations (Fig S2d). Hard carbon is often described as a mixture of curved and entangled graphene and fullerene-like structures.[1-4] This turbostatic network creates nanopores in various sizes and number distributions.[5] The graphene sheets are highly defective, containing single and double vacancies and heteroatom contamination.[2,3,6,7] Experimental structural evaluation of the nanostructure[5] has shown a range of nanopore sizes (0.6 to 1.6 nm), with the distribution maximum depending on the annealing temperature. Thus curved and misaligned nanopore configurations containing exposed edges and defects with realistic nanopore sizes (1.2 nm for "vertical edge to plane," "0.85nm for the power button," and 0.6 to 1.4 nm for the "wedge pore") are good approximations to study the environment of the interaction in the pore and, more specifically, capture the slope to plateau transitional behavior.

The pore environment is challenging computationally. For this reason, the carbon matrix was frozen in the "vertical edge to plane" and "power button" and initially on "wedge pore" configurations. In a subsequent relaxation step, the most stable points of the "wedge pore" configuration were relaxed with no restraints so that the pore could adjust towards the lowest energy configuration. We report both the frozen matrix and fully relaxed results as they provide an intuition of the behavior of a nonplastic pore, a buried nanopore, that might not be that flexible to expand. Further, the frozen pores are capable of capturing the fundamental interaction between the metal-carbon interfaces, additionally giving us the relative filling fraction of the three alkali metals in specific pore sizes. Knowing from the first part of our investigation and the cited literature how alkali metals behave in defective environments (edges and vacancies give rise to the sloping voltage region), we design the pore environments as simply as possible to avoid the computational cost. For this reason, the "vertical edge to plane" "power button"

and "wedge pore" contain zero, zero, and one vacancy, respectively. Including more vacancies will result in more points in the sloping voltage profile. Lithiation, sodiation, and potasiation were simulated by inserting Li, Na, and K in competing configurations. The number of configurations tested is affected by the number of ions we can fill the wedge pore. Once all the pore space is filled with alkali metals in realistic close-packed distances, we tested a few super-packed configurations and experienced a sharp energy rise. Thus, we considered as the completely filled reference phase the one before experiencing the energy rise. In total, 91 Li, 41 Na, and 21 K are the number of atoms for the lithiated, sodiated, and potentiated wedge pores.

Finally, some stacking of the graphene sheets is observed, usually ranging from two to five layers with expanded interlayer distances compared to graphite.[8-10] Intercalation configurations were also studied (FigS2 C) for various interlayer ranges, chosen to include realistic distances measured experimentally and calculated computationally.[8-11]

Spin polarization

Spin polarization was tested for the different environments. In most configurational environments, we find that it does not affect the qualitative and quantitative calculations (reaction mechanisms and trends) and, thus, marginally affects the calculated voltages (Figure S2a,b,c Table S1). However, spin polarization produces a quantitative difference worth noting in the presence of edges (Figure S2d). Utilizing hundreds of DFT calculations covering a broad range of environments, we chose a balanced calculation scheme where the bulk of the calculations(convex hull relaxations) does not include spin. The most stable relaxed structures were re-relaxed in a subsequent step with spin polarization.

Table S1: Effect of spin polarization on non-edge environments

**Environment**	concentration	dV(spin-nonspin) (V)
a. Defect plane (SV) – Na	1 atom	0.007
b. Defect plane (DV) – Na	1 atom	0.013
c. Defect plane (DV) – Li	1 atom	0.017
Defect plane, relative difference Na/Li position		0.004
d. Defect plane – Na	16 atoms (layering)	0.007

e. Wedge pore plateau (Na)	45 Na	0.018
f. Wedge pore plateau (Li)	91 Li	0.013
g. Wedge pore plateau (K)	21 K	0.028
Wedge pore, relative difference Na/Li position		0.012

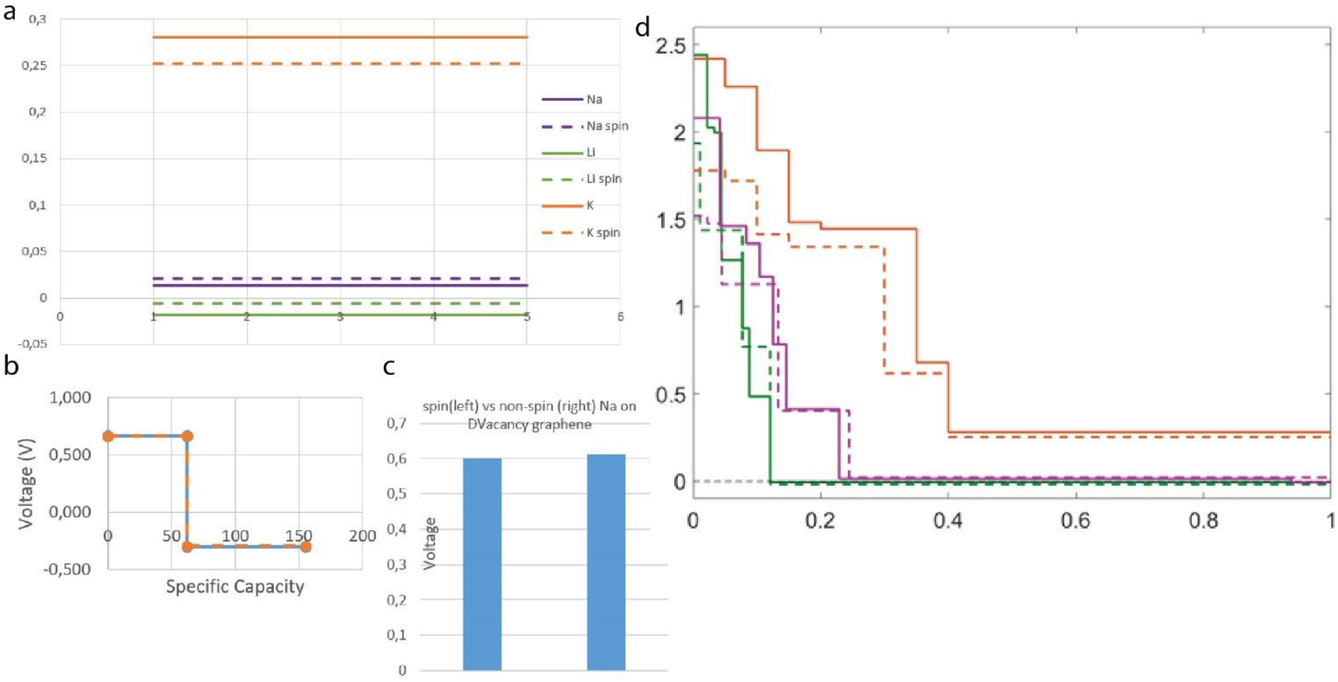

Figure S2. Example visuals of how the voltage lines shift with the inclusion of spin-polarized calculations of the a) wedge-pore, b) formation voltage of pyramid on double vacancy, c) double vacancy defect plane, d) Example of spin polarization effect on edges. Dotted lines show the spin-polarized calculations, and solid lines show the non-spin calculations. Spin polarization on the edge environments drags down the initial points of the voltage profile.

Supplementary Information B

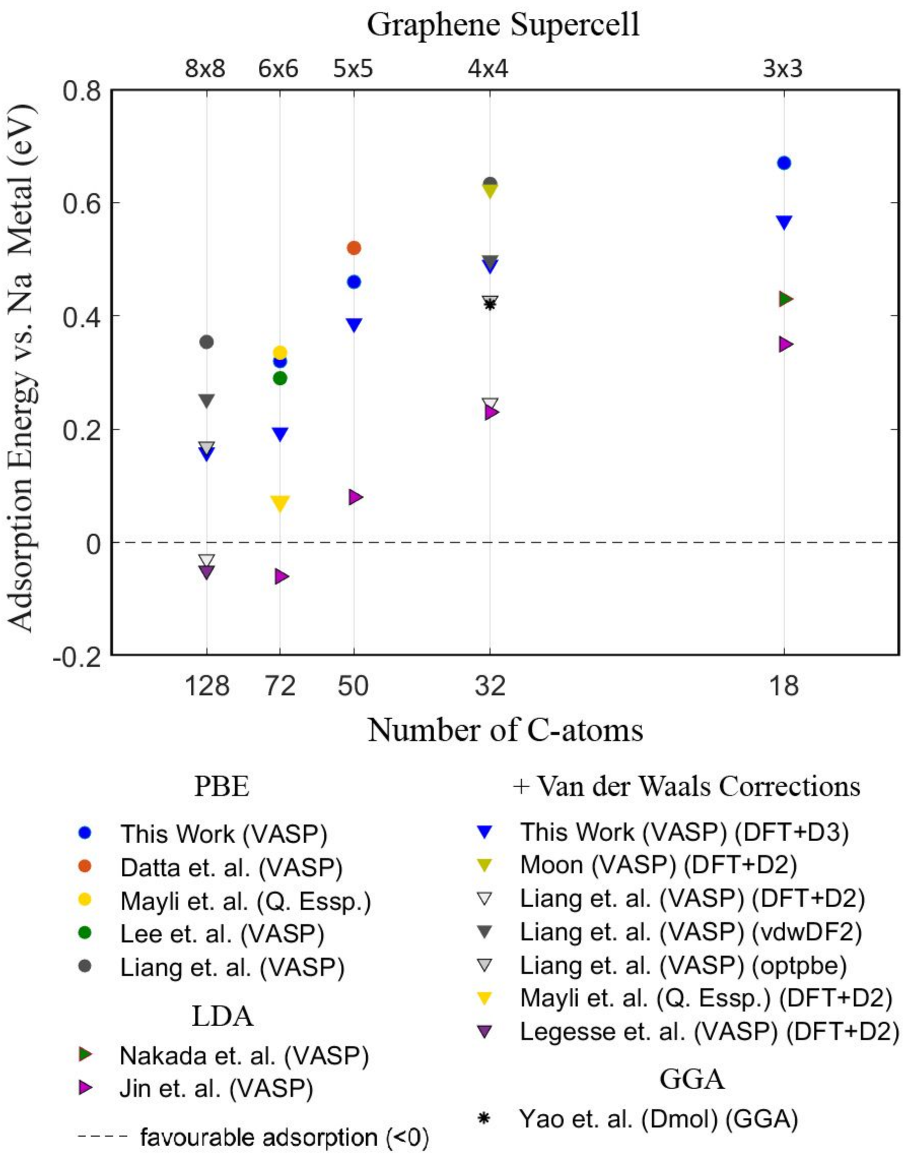

Figure S2 Adsorption energy vs. supercell size (specific capacity) of one Na atom on pristine graphene compared with results obtained from the literature for various functionals and dispersion error-correction methods.[12-19]. A slight offset is introduced in the plot where scatter points are too close to each other. One Na on the 8x8, 6x6, 5x5, 4x4, and 3x3 supercells responds to 18, 21, 45, 70, 124, and mAh/g, respectively.

Supplementary Information C

Surface energies of Li/Na/K

Surface energy is defined as the excess energy at the surface of a crystalline material compared to the bulk, quantifying the energy required to build an area of the particular surface. In agreement with previous experimental and computational studies[20-22], we find that Li surfaces consistently pose a more significant energy penalty than Na and K. This indicates that Li nanoclusters with exposed surfaces will be less favorable than Na and K nanoclusters.

Metal	**Surface Energy of the (110) (j/m^2)**	**Experimental (j/m^2)**	**Other Theoretical Results (j/m^2)**	
Li	0.571	0.522[9]	0.585[21]	0.556[22]
Na	0.320	0.261[9]	0.247[21]	0.253[22]
K	0.152	0.145[9]	0.137[21]	0.135[22]

Method	**This work**	**Reference 8[21]**	**Reference 9[22]**
functional	PBE	PBE	FCD
corrections	DFT-D3	-	-
cut-off	500eV	400eV	-
smearing	Methfessel-Paxton	Methfessel-Paxton	Methfessel-Paxton

Our computational results reproduce the experimentally and computationally observed alkali-ion surface energy trend ($\gamma_{Li}>\gamma_{Na}>\gamma_{K}$).[21] We focus on the (110) surface. This is the lowest energy surface for Na and K having the highest facet contribution probability.[21] Even though for Li the (100) surface appears to have slightly lower energy (0.541 j/m^2), the calculated work function, which quantifies the difficulty of the electron to escape the plane, is consistently calculated as larger for the (110) surface for all the alkali-ions.[21-22]

Supplementary Information D

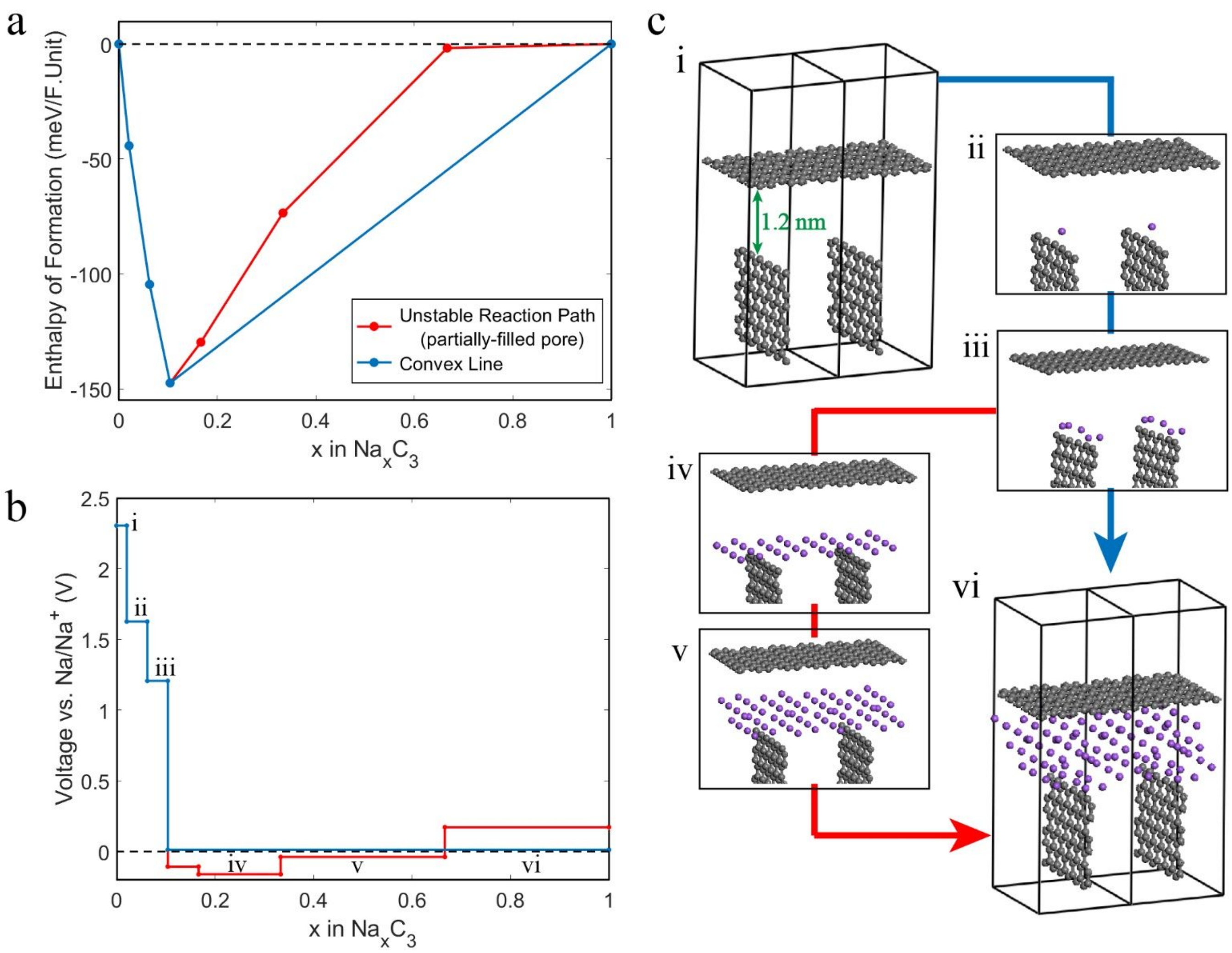

Figure S3 a) convex hull and b) voltage profile of sodiation of the vertical "edge to plane" configuration. c) configurations throughout the sodiation of the vertical "edge to plane" configuration. All figures include both the unfavorable sodiation way (red path) and the favorable first-order phase transition way (blue path).

Our calculations indicate that the system prefers to go from step iii directly to the completely filled pore (step vi) as intermediate partially filled pores have exposed surfaces and thus plunge the voltage below 0V.

Supplementary Information E

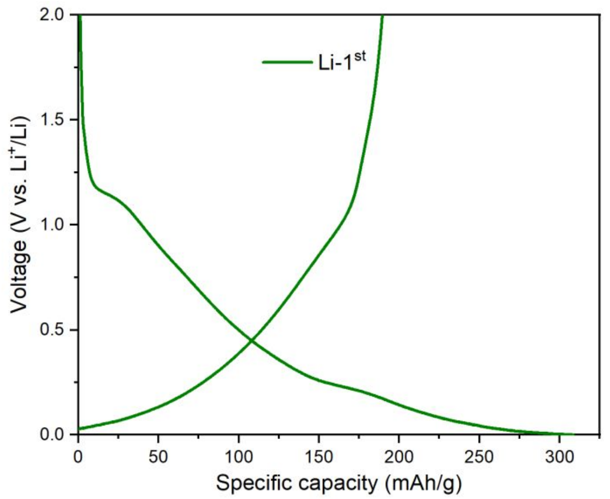

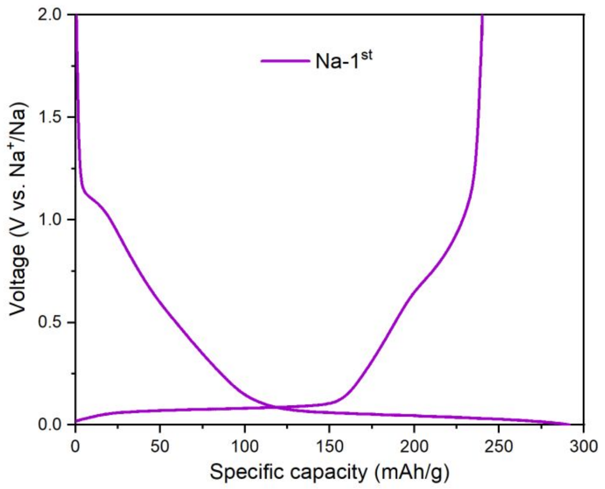

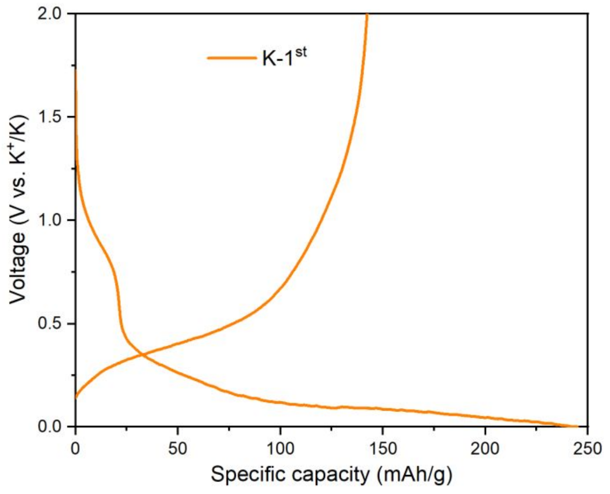

Figure S4 First cycle of galvanostatic discharge curves in Li, Na and K half-cells, respectively

Supplementary Information F

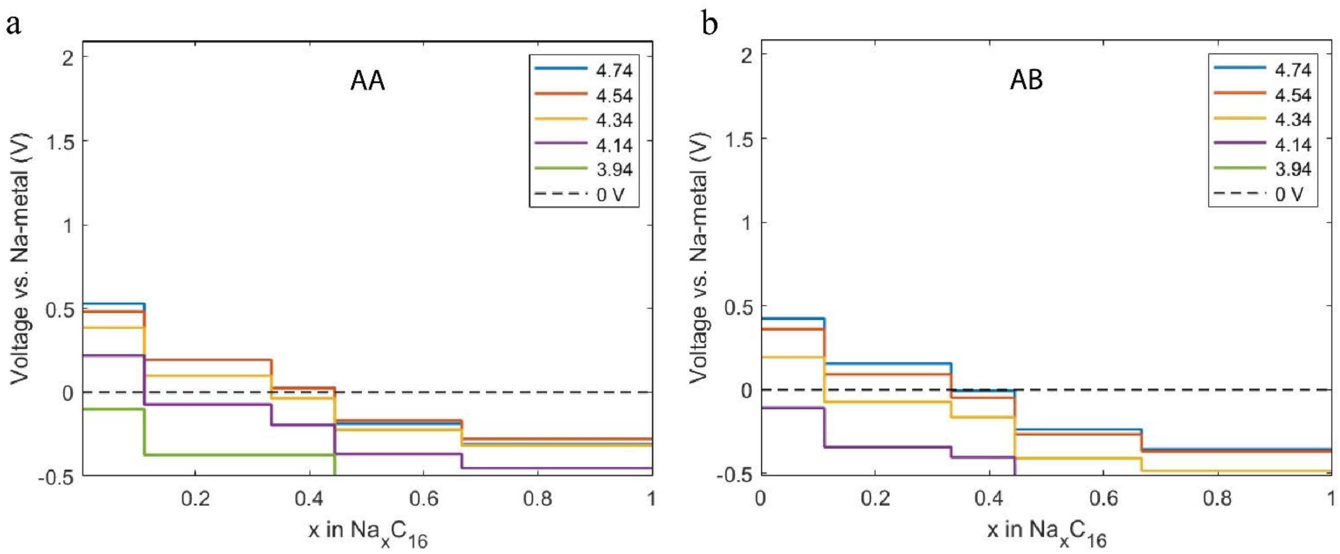

Figure S5 First cycle of galvanostatic discharge curves in Li and Na half-cells, respectively

References

(1) Dou, X. W.; Hasa, I.; Saurel, D.; Vaalma, C.; Wu, L. M.; Buchholz, D.; Bresser, D.; Komaba, S.; Passerini, S. Hard carbons for sodium-ion batteries: Structure, analysis, sustainability, and electrochemistry. Materials Today 2019, 23,

(2) Edwards, A.S. I.; Marsh, H. Introduction of carbon science, Butterworths, 1989.

(3) Li, Z. F.; Bommier, C.; Sen Chong, Z.; Jian, Z. L.; Surta, T. W.; Wang, X. F.; Xing, Z. Y.; Neuefeind, J. C.; Stickle, W. F.; Dolgos, M.; Greaney, P. A.; Ji, X. L. Mechanism of Na-Ion Storage in Hard Carbon Anodes Revealed by Heteroatom Doping. Advanced Energy Materials 2017, 7, 1602894.

(4) Stevens, D. A.; Dahn, J. R. An in situ small-angle X-ray scattering study of sodium insertion into a nanoporous carbon anode material within an operating electrochemical cell. Journal of Electrochemical Society, 2000, 147, 4428-4431.

(5) Kubota, K.; Shimadzu, S.; Yabuuchi, N.; Tominaka, S.; Shiraishi, S.; Abreu-Sepulveda, M.; Manivannan, A.; Gotoh, K.; Fukunishi, M.; Dahbi, M.; Komaba, S. Structural Analysis of Sucrose-Derived Hard Carbon and Correlation with the Electrochemical Properties for Lithium, Sodium, and Potassium Insertion. Chemistry of Materials 2020, 32, 2961-2977.

(6) Robinson, J. T.; Zalalutdinov, M. K.; Cress, C. D.; Culbertson, J. C.; Friedman, A. L.; Merrill, A.; Landi, B. J. Graphene Strained by Defects. ACS Nano 2017, 11, 4745-4752.

(7) Harris, P. J. F. New perspectives on the structure of graphitic carbons. Critical Reviews in Solid State and Materials Sciences, 2005, 30, 235-253.

(8) Stevens, D. A.; Dahn, J. R. An in situ small-angle X-ray scattering study of sodium insertion into a nanoporous carbon anode material within an operating electrochemical cell. Journal of Electrochemical Society, 2000, 147, 4428-4431.

(9) Qiu, S.; Xiao, L. F.; Sushko, M. L.; Han, K. S.; Shao, Y. Y.; Yan, M. Y.; Liang, X. M.; Mai, L. Q.; Feng, J. W.; Cao, Y. L.; Ai, X. P.; Yang, H. X.; Liu, J. Manipulating Adsorption-Insertion Mechanisms in Nanostructured Carbon Materials for High-Efficiency Sodium Ion Storage. Advanced Energy Materials, 2017, 7, 1700403.

(10) Lin, X. Y.; Liu, Y. Z.; Tan, H.; Zhang, B., Advanced lignin-derived hard carbon for Na-ion batteries and a comparison with Li and K ion storage. Carbon, 2020, 157, 316-323.

(11) Olsson, E., Cottom, J., Cai, Q. Defects in Hard Carbon: Where Are They Located and How Does the Location Affect Alkaline Metal Storage? Small 2021, 17, 2007652.

(12) Nakada, K.; Ishii, A. DFT Calculation for Adatom Adsorption on Graphene. Graphene Simulation 2011, 3-20.

(13) Jin, K. H.; Choi, S. M.; Jhi, S. H. Crossover in the adsorption properties of alkali metals on graphene. Physical Review B 2010, 82 (3).

(14) Moon, H. S.; Lee, J. H.; Kwon, S.; Kim, I. T.; Lee, S. G. Mechanisms of Na adsorption on graphene and graphene oxide: density functional theory approach. Carbon Letters 2015, 16 (2), 116-120.

(15) Yao, L. H.; Cao, M. S.; Yang, H. J.; Liu, X. J.; Fang, X. Y.; Yuan, J. Adsorption of Na on intrinsic, B-doped, N-doped and vacancy graphenes: A first-principles study. Computational Material Science 2014, 85, 179-185.

(16) Sun, X. L.; Wang, Z. G.; Fu, Y. Q. Adsorption and diffusion of sodium on graphene with grain boundaries. Carbon 2017, 116, 415-421.

(17) Datta, D.; Li, J. W.; Shenoy, V. B. Defective Graphene as a High-Capacity Anode Material for Na- and Ca-Ion Batteries. ACS Applied Material and Interfaces 2014, 6 (3), 1788-1795.

(18) Malyi, O. I.; Sophia, K.; Kulish, V. V.; Tan, T. L.; Manzhos, S.; Persson, C. A computational study of Na behavior on graphene. Applied Surface Science 2015, 333, 235-243.

(19) Legesse, M.; El Mellouhi, F.; Bentria, E.; Madjet, M. E.; Fisher, T. S.; Kais, S.; Alharbi, F. H. Reduced work function of graphene by metal adatoms. Applied Surface Science 2017, 394, 98-107.

(20) Tyson, W. R.; Miller, W. A., Surface Free-Energies of Solid Metals - Estimation from Liquid Surface-Tension Measurements. Surface Science 1977, *62* (1), 267-276.

(21) Wang, J.; Wang, S. Q., Surface energy and work function of fcc and bcc crystals: Density functional study. Surface Science 2014, 630, 216-224.
(22) Vitos, L.; Ruban, A. V.; Skriver, H. L.; Kollar, J., The surface energy of metals. Surface Science 1998, 411 (1-2), 186-202.
